# Phenotypic and genotypic assessment of antimicrobial and biocide resistance in enterobacteria from anaerobic digestion of dairy waste: implications for environmental and public health

**DOI:** 10.1007/s11274-026-05319-9

**Published:** 2026-10-08

**Authors:** Mateus Rodrigues Alves, Nicolly Soares Ferreira, Max Sulivan Fontes, Mariana Fabri Lima, Marcelo Henrique Otenio, Mariana Drummond Costa Ignacchiti, Juliana Alves Resende

**Affiliations:** 1 Graduate Program in Veterinary Sciences, Center for Agricultural Sciences and Engineering - CCAE, Federal University of Espírito Santo - UFES, Alto Universitario, no number, Guararema, Alegre, ES 29500-000 Brazil; 2 Graduate Program in Pharmaceutical Sciences, Center of Health Sciences, Federal University of Espírito Santo (UFES), Vitoria, Espírito Santo State 29043-900 Brazil; 3 Department of Pharmacy and Nutrition, Federal University of Espírito Santo - UFES, Alegre, ES 29500-000 Brazil; 4 Embrapa Dairy Cattle - Brazilian Agricultural Research Corporation, Juiz de Fora, Brazil

**Keywords:** Anaerobic digestion, Antimicrobial resistance, Biocide, Dairy cattle, Enterobacterales

## Abstract

Antimicrobial-resistant bacteria in agricultural waste represent a critical One Health concern. Anaerobic digestion of livestock waste is widely promoted; however, the fate of antimicrobial resistance and biocide tolerance in full-scale systems remains unclear, constraining safe digestate reuse. This study assessed phenotypic and genotypic resistance in *Escherichia coli-like* enterobacteria isolated from effluent and biofertilizer in a full-scale dairy anaerobic digestion system. A total of 173 isolates were screened for antimicrobial resistance genes (ARGs), antimicrobial susceptibility, multidrug resistance (MDR), and biocide inhibition-zone responses. No diarrheagenic virulence genes were detected. At least one screened ARG was detected in 91.0% of effluent and 83.3% of biofertilizer isolates. *qacE∆1* was the most frequent marker and occurred significantly more often in effluent (70.8%) than biofertilizer isolates (44.0%; q = 0.00291). Phenotypic resistance to at least one antimicrobial was observed in 27.0% and 34.5% of effluent and biofertilizer isolates, respectively, while MDR occurred in 2.2% and 9.5% (*p* = 0.0521). *tet(a)* carriage was strongly associated with tetracycline resistance (OR = 20.9; q < 0.001). H_2_O_2_ inhibition zones were significantly smaller among biofertilizer isolates, whereas Lactic Acid (LA) yielded 6-mm measurements in all isolates and in the reference strain. These findings demonstrate that anaerobic digestion does not fully eliminate culturable resistant enterobacteria and underscore the need for integrated resistance monitoring in dairy-waste management.

**Supplementary Information:**

The online version contains supplementary material available at https://doi.org/10.1007/s11274-026-05319-9.

## Introduction

Global population growth increases the demand for food, impacting dairy farming, which provides essential raw materials for various food products (Erdaw, 2023). Brazil is one of the world’s leaders in animal production, with a vast cattle herd aimed at both domestic consumption and exportation. According to data from the Brazilian Ministry of Agriculture and Livestock (MAPA, 2023), Brazil ranks third globally in cattle herd size. However, the expansion of cattle farming poses significant challenges, including pressure on natural resources such as water and land, and the need to enhance efficiency and production sustainability due to the high volume of waste generated by this activity (Rahut et al. 2024).

Animal production waste, such as feces and urine, represents a large amount of biomass, and its recycling is essential for both economic and environmental reasons. The use of anaerobic biodigesters on dairy farms offers a solution for managing this waste, converting it into biofertilizer and biogas. The biodigester promotes the decomposition of organic matter in the absence of oxygen, producing valuable resources for soil fertilization and renewable energy. Within circular production systems, biodigesters play a strategic role in closing nutrient and energy loops on dairy farms. However, emerging evidence suggests that antimicrobial resistance and biocide tolerance may represent unintended sustainability trade-offs of this technology. If resistant bacteria or resistance determinants persist or are enriched during digestion, the environmental benefits of nutrient recycling may be partially offset by microbiological risks (Wang et al. 2025; Resende et al. 2014a, b).

*Escherichia coli*, a resident bacterium of the intestinal tract of animals and humans, is often used as an indicator of fecal contamination and as a model organism for studying antimicrobial resistance due to its ability to transfer antimicrobial resistance genes (ARGs) through binary fission as well as transduction, transformation, and conjugation mechanisms. The increase in antimicrobial-resistant *E. coli* represents a significant threat to public health, especially when these bacteria are released into the environment through improperly treated effluents (Furlan et al. 2020).

Antimicrobial resistance is a global concern, recognized as an emerging threat by the World Health Organization (WHO) (Ahmed et al. 2024). The spread of ARGs in agricultural environments, particularly in waste management systems, can exacerbate this problem. Previous studies have demonstrated that although anaerobic digestion systems effectively reduce the overall microbial load, they may not fully eliminate pathogenic microorganisms or antimicrobial resistance genes present in bovine waste (Pham et al. 2024; Li et al. 2023; Burch et al. 2022).

Most work on antimicrobial resistance during anaerobic digestion has focused either on community-level ARG abundance or on phenotypic characterization, while full-scale studies that connect targeted ARG carriage, antimicrobial susceptibility, and biocide inhibition responses within the same culturable isolates remain comparatively limited (Burch et al. 2022; Lu et al. 2021; Wallace et al. 2018). This distinction is biologically relevant because community-level changes in gene abundance do not identify which viable enterobacteria remain recoverable after treatment, and the detection of a resistance gene in an isolate does not necessarily result in functional phenotypic resistance. The unanswered question addressed here is therefore not simply whether resistance markers occur in dairy-waste treatment systems, but whether selected ARGs and resistance phenotypes co-occur at the isolate level in culturable enterobacteria recovered from two points of a full-scale anaerobic digestion process. By integrating targeted ARG and diarrheagenic-marker screening with antimicrobial susceptibility, biocide inhibition-zone measurements, and formal genotype-phenotype association analyses, this study provides an isolate-level assessment that complements community-level surveillance.

Accordingly, this study aimed to characterize phenotypic and targeted genotypic resistance profiles of culturable enterobacterial isolates with presumptive *E. coli*-like characteristics recovered from two process locations of a full-scale anaerobic digester treating dairy cattle manure: the biodigester inlet (effluent from the production unit) and the digestate reservoir (treated material referred to here as biofertilizer). We asked whether (i) frequencies of the screened ARGs differed between the two matrices; (ii) antimicrobial resistance and phenotypic multidrug resistance differed between matrices; (iii) biologically matched resistance markers were statistically associated with their corresponding phenotypes; and (iv) biocide inhibition-zone responses differed between matrices. By addressing these questions, this research aims to contribute to the understanding of resistance dynamics in livestock waste treatment systems and to inform safer practices regarding the use of biodigester effluents in agriculture.

## Materials and methods

### Sample collection and isolation of presumptive *E. coli*-like enterobacteria

Samples were collected from a semi-continuous, full-scale anaerobic digester located at the experimental dairy farm of Embrapa Gado de Leite (Coronel Pacheco, Minas Gerais, Brazil; 21°33′58″S; 43°15′21″W; 445 m altitude). The climate of the region is classified as humid subtropical (Cwa) according to the Köppen and Geiger scale (Alvares et al. 2013). The system treats dairy cattle manure through a multi-stage process, including preliminary solid separation, anaerobic digestion with sludge recirculation, and final storage of the treated effluent (biofertilizer) (Mendonça et al. 2017; Nascimento et al. 2020).

The biodigester operates at ambient temperature and has a useful capacity of 540 m³, with a hydraulic retention time (HRT) of 32 days (± 6.4). The treatment train includes an equalization tank, centrifugal screening/solid separation, sediment removal, a plug-flow anaerobic digester with sludge recirculation, and a final sedimentation/biofertilizer pond with an HRT of approximately 7 days. The substrate is generated during floor washing in the semi-confined free-stall system of the Genizinha dairy farm. Treated liquid from the final pond is reused for floor washing and therefore re-enters the treatment train with newly generated cattle wastewater (Nascimento et al. 2020). Physicochemical measurements obtained concurrently with the sampling events are summarized in Supplementary Table S1. The system operated at average ambient temperatures of 24.5 °C in March and 18.5 °C in June, with near-neutral pH in both matrices. Alkalinity, acidity, intermediate/partial alkalinity (IA/PA) ratio, and total solids were also determined.

The experimental campaign comprised 12 samples of 500 mL collected from the two process locations: six effluent samples and six digestate/biofertilizer samples. Sampling was performed as six paired weekly collection events, with three paired collections in March 2022 and three in June 2022. In the present study, sampling period was not used as an analytical factor; isolates from the two collection periods were pooled and compared according to process matrix (effluent versus biofertilizer). Samples were collected into sterile bottles, transported in an isothermal container, and processed at the Rumen Microbiology Laboratory of Embrapa Dairy Cattle, Juiz de Fora, MG.

For microbiological processing, a 1-mL aliquot from each independent sample was serially diluted in sterile 0.9% NaCl solution to 10^− 8^ before culture-based isolation. For the isolation of enterobacteria suggestive of *E. coli*, Petrifilm™ plates (Petrifilm™ EC 6404, 3 M™) were used. The procedure followed the manufacturer’s instructions. The presumptive identification of *E. coli* colonies on Petrifilm™ is evidenced by the presence of blue colonies with gas bubble formation. The colonies suggestive of *E. coli* were selected and cultured on Eosin Methylene Blue (EMB) agar (Himedia) at 35 °C for 24 h. Colonies with a characteristic metallic green sheen, indicative of *E. coli*, were inoculated on Triple Sugar Iron (TSI) agar (Himedia) and Brain Heart Infusion (BHI) agar (Himedia) for subsequent analysis of their morphological and staining characteristics by Gram staining. Individual colonies selected through this procedure were archived as separate isolates for subsequent analyses.

### Genomic DNA extraction

For the extraction of genomic DNA, the bacterial isolates were cultured in BHI broth for 24 h at 35 °C. Genomic DNA was then extracted from the resulting bacterial cultures using a commercial kit, following the manufacturer’s instructions (PureLink™ Genomic DNA Mini Kit, Invitrogen).

The DNA was quantified using a Nanodrop spectrophotometer (Thermo Scientific) and diluted to a concentration of 10 ng/µL in ultrapure, RNase- and DNase-free water (Invitrogen). The DNA was stored in a freezer at − 20 °C for later use as a template DNA in PCR.

### Molecular characterization by Polymerase Chain Reaction (PCR)

A PCR assay targeting the 16 S rRNA gene sequence, previously used as a screening tool for isolates presumptively belonging to as *E. coli*, was applied as an additional molecular screening step (Charaya et al. 2015). Because 16 S rRNA-based approaches have limited discriminatory power within the *E. coli*/*Shigella* complex, species-level identification was not considered definitive in this study (Ragupathi et al. 2018). Therefore, isolates fulfilling the culture, biochemical, and PCR screening criteria are referred to throughout as culturable enterobacterial isolates with presumptive *E. coli*-like characteristics.

For screening of diarrheagenic *E. coli*-associated virulence markers, multiplex PCR assays were performed using primers and conditions established by Aranda et al. (2004). The targets were eae and bfpA for EPEC, CVD432 for EAEC, LT and ST for ETEC, stx1 and stx2 for STEC, and ipaH for EIEC/*Shigella* (Supplementary Table S2).

For the amplification of resistance genes, PCR analyses were conducted with specific primers (Invitrogen Custom Primers) for the amplification of genes encoding resistance markers to beta-lactams, *bla_TEM* and *ampC;* sulfonamides, *sul1*; aminoglycosides, *aacA-aphD*; chloramphenicol/florfenicol, *floR;* tetracyclines, *tet(a)*, and quaternary ammonium compounds, *qacEΔ1*. The amplification conditions followed those described in the literature (Supplementary Table S3).

All PCR reactions were performed in a final volume of 50µL, containing ~ 20 ng/µL of DNA, and the primer volume as per the reference. The reactions were carried out in an automated thermocycler (Dlab, TC1000-G). The amplicons were visualized in a photodocumenter (Gel DocTM XR+, Bio-Rad) after electrophoresis on a 1.5% agarose gel (80 V, 500 mA, 150 W for 45 min).

The amplicons were stained with SYBR Safe DNA Gel Stain (Invitrogen). As a molecular weight standard, a 1Kb DNA ladder marker (Life Technologies Inc.) was used. The negative control for the reactions consisted of adding RNase- and DNase-free water (Invitrogen) in place of the template DNA, and as a positive control, samples from the Applied Microbiology Group (GrAM) collection, stored at the Clinical Analysis Laboratory (LAC) of UFES – Campus Alegre.

### Antimicrobial and biocide inhibition-zone testing

Antimicrobial susceptibility was determined using the disk diffusion technique, according to the Clinical & Laboratory Standards Institute (CLSI, 2025). The antimicrobials evaluated were: AMC - Amoxicillin-Clavulanic Acid (30 µg); TET - Tetracycline (30 µg); CAZ - Ceftazidime (30 µg); GEN - Gentamicin (10 µg); CLO - Chloramphenicol (30 µg); MER - Meropenem (10 µg); and LVX - Levofloxacin (5 µg). The plates were incubated at 35 ± 2 °C for 18–20 h. Inhibition zones were measured and isolates were classified as susceptible (S), intermediate (I), or resistant (R) using the applicable CLSI breakpoints. Tests were performed in triplicate, with *E. coli* ATCC 25,922 as quality-control strain. For the study-level summary, phenotypic multidrug resistance (MDR) was defined as resistance (R) to agents representing at least three antimicrobial categories among those tested; intermediate results were reported separately and were not counted as resistance.

Biocide responses were evaluated using an agar disk diffusion assay. The compounds tested were: 2% Lactic Acid (LA) (Rioquímica), 0.28% benzalkonium chloride (BAC) (Rioquímica), 3% hydrogen peroxide (H_2_O_2_) (Rioquímica), and 1% chlorhexidinedigluconate (CHX) (Rioquímica). These compounds and concentrations were chosen due to reflect those commonly applied in disinfecting materials or/and sanitizing teats during the milking process. Bacterial suspensions were prepared in sterile saline solution (0.9% NaCl), adjusted to 0.5 on the McFarland scale (10^8^ CFU/mL), and then spread over the surface of plates containing Mueller Hinton agar (MHA) (Himedia). Sterile filter paper discs (6 mm) were impregnated with 5 µL of the selected biocides and placed on the culture medium; the plates were incubated at 35 °C for 24 h. After incubation, the inhibition zones were measured. The *E. coli* ATCC 25,922 reference strain was tested under the same conditions.

### Statistical analysis

Statistical analyses were performed using two-sided tests with a significance level of 5% (α = 0.05). Frequencies of individual ARGs, antimicrobial resistance phenotypes, carriage of at least one or three or more ARGs, resistance to at least one antimicrobial, and phenotypic MDR were compared between effluent and biofertilizer isolates using Fisher’s exact test. P-values from multiple comparisons were adjusted using the Benjamini–Hochberg false-discovery-rate procedure.

Genotype–phenotype associations were evaluated using Fisher’s exact test, odds ratios (OR), and Cohen’s kappa coefficient for the corresponding resistance gene–antimicrobial pairs. Biocide inhibition-zone diameters were compared between matrices using the Mann–Whitney U test, with Benjamini–Hochberg adjustment for multiple comparisons. Lactic acid was excluded from inferential analysis because all inhibition-zone measurements were 6 mm. Exact p-values and adjusted q-values are presented in Supplementary Tables S4 – S6.

## Results

### Isolation and screening of presumptive *E. coli*-like enterobacteria

A total of 180 lactose-fermenting enterobacterial isolates with phenotypic characteristics suggestive of *E. coli* were included, comprising 90 isolates from the effluent and 90 from the biofertilizer. Initial identification was performed by assessing β-glucuronidase activity using chromogenic medium (Petrifilm™ EC 6404, 3 M™). Additional biochemical assays and growth on selective media were conducted following protocols described by Bird et al. (2020).

The 16 S rRNA-targeted PCR screening assay yielded amplicons in 96.1% (173/180) of the isolates: 89 from the effluent and 84 from the biofertilizer. Given the taxonomic limitation of the assay, these 173 isolates are described hereafter as PCR-positive presumptive *E. coli*-like enterobacteria.

### Detection of diarrheagenic virulence-associated markers

None of the 173 isolates was positive for the investigated virulence-associated markers of EPEC, STEC, ETEC, EAEC, or EIEC/*Shigella*.

### Molecular detection of ARGs

**Fig. 1:**
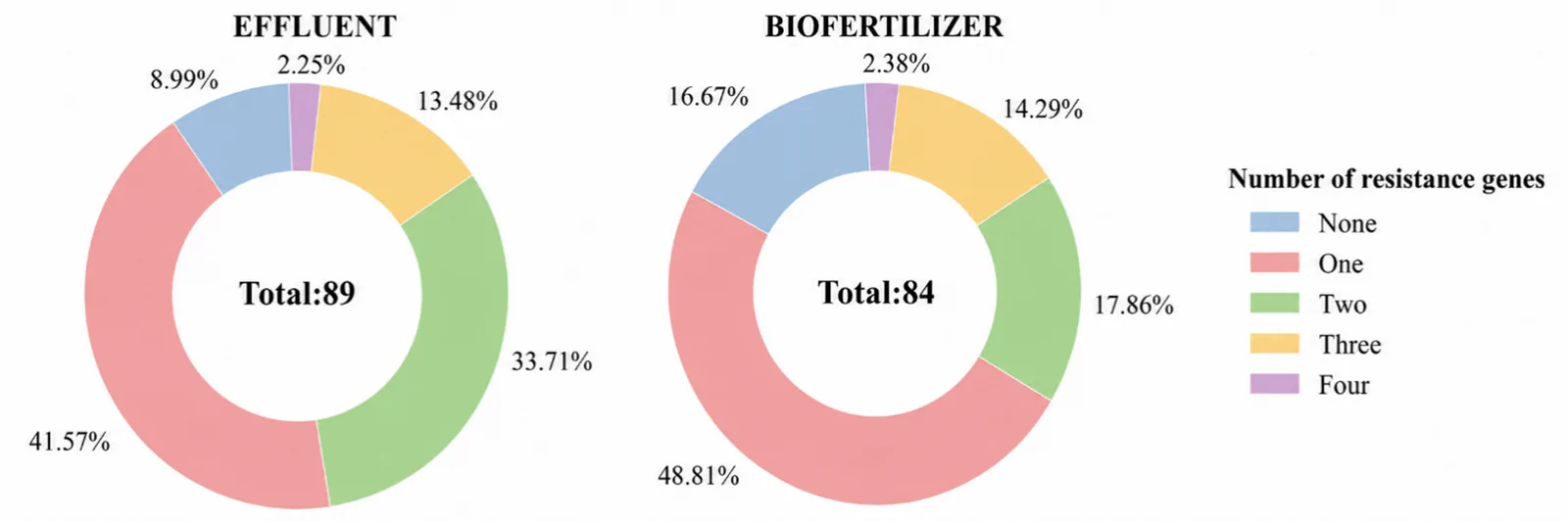
Distribution of the number of screened resistance genes per *E. coli*-like enterobacterial isolate from anaerobic digester influent and biofertilizer

Among the 173 presumptive *E. coli*-like enterobacterial isolates, at least one of the seven screened antimicrobial resistance genes (ARGs) was detected in 91.0% of effluent isolates (81/89) and 83.3% of biofertilizer isolates (70/84), with no significant difference between matrices (Fisher’s exact test, *p* = 0.171). Twenty-eight isolates carried three or more screened ARGs, including 15.7% (14/89) of effluent and 16.7% (14/84) of biofertilizer isolates (*p* = 1.000) (Fig. 1).

**Fig. 2:**
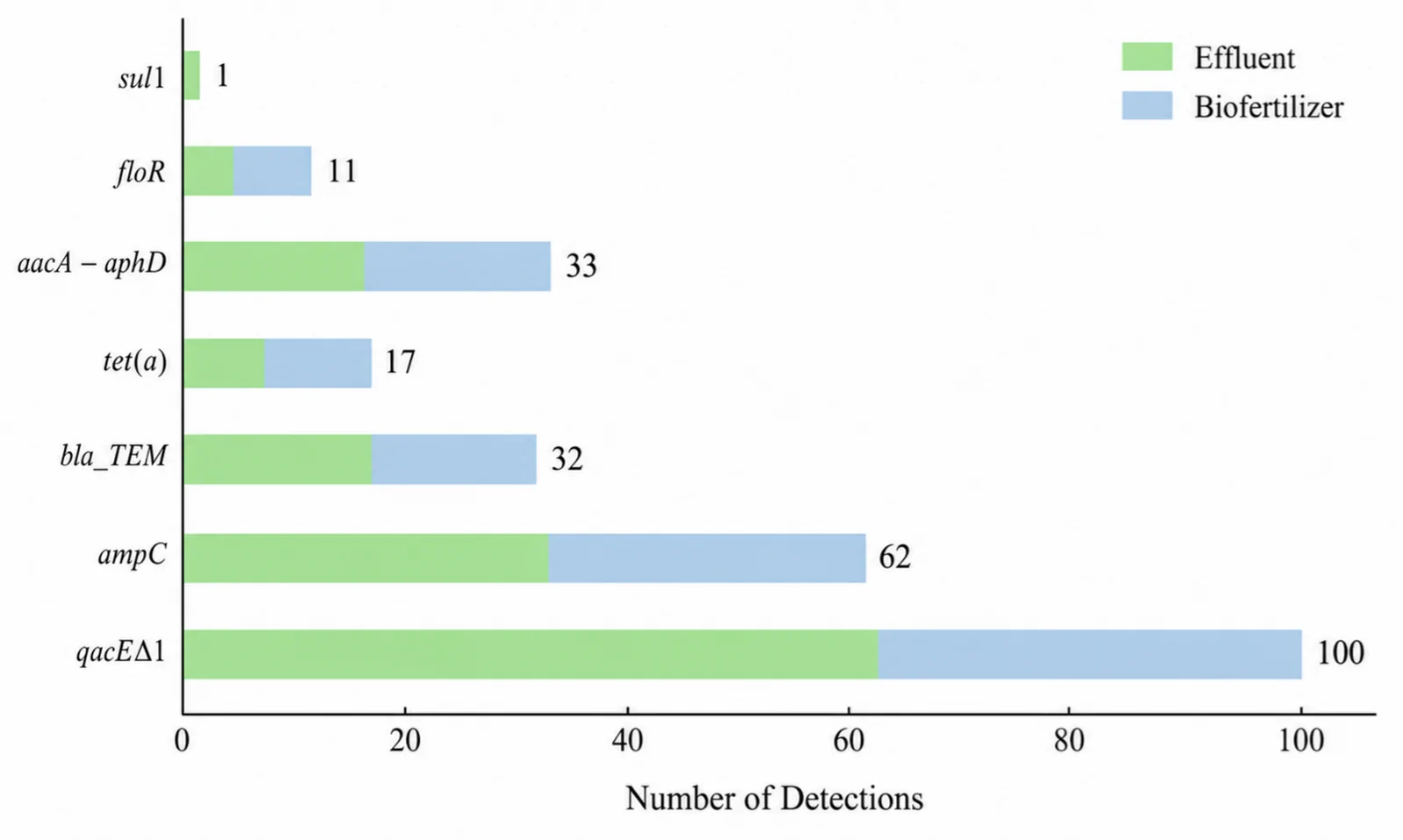
Absolute frequency of selected resistance-gene detection in *E. coli*-like enterobacterial isolates from anaerobic digester influent and biofertilizer

The distribution of individual ARGs is shown in Fig. 2. The most frequently detected gene was *qacE∆1*, identified in 57.8% (100/173) of all isolates. This gene occurred in 70.8% (63/89) of effluent isolates and 44.0% (37/84) of biofertilizer isolates, representing the only ARG with a significant difference between matrices (Fisher’s exact test, *p* = 0.000415; OR = 3.08; q = 0.00291). The *ampC* gene was the second most prevalent marker, detected in 35.8% (62/173) of isolates, including 37.1% (33/89) from the effluent and 34.5% (29/84) from the biofertilizer. The *aacA-aphD* gene was detected in 19.1% (33/173) of isolates, corresponding to 18.0% (16/89) of effluent and 20.2% (17/84) of biofertilizer isolates. Other ARGs detected included *bla_TEM* (18.5%, 32/173 total sample; 19.1% [17/89] effluent and 17.8% [15/84] biofertilizer), *tet(a)* (9.8%, 17/173 total sample; 7.8% [7/89] effluent and 11.9% [10/84] biofertilizer), and *floR* (6.35%, 11/173 total sample; 4.5% [4/89] effluent and 8.3% [7/84] biofertilizer). The *sul1* gene, was found in only a single effluent isolate (0.6% of all isolates). No significant differences between matrices were observed for *ampC*, *aacA-aphD*, *bla_TEM*, *tet(a)*, *floR*, or *sul1* after multiple-testing correction (Fig. 2; Supplementary Table S4).

### Phenotypic antimicrobial resistance and MDR patterns

**Fig. 3:**
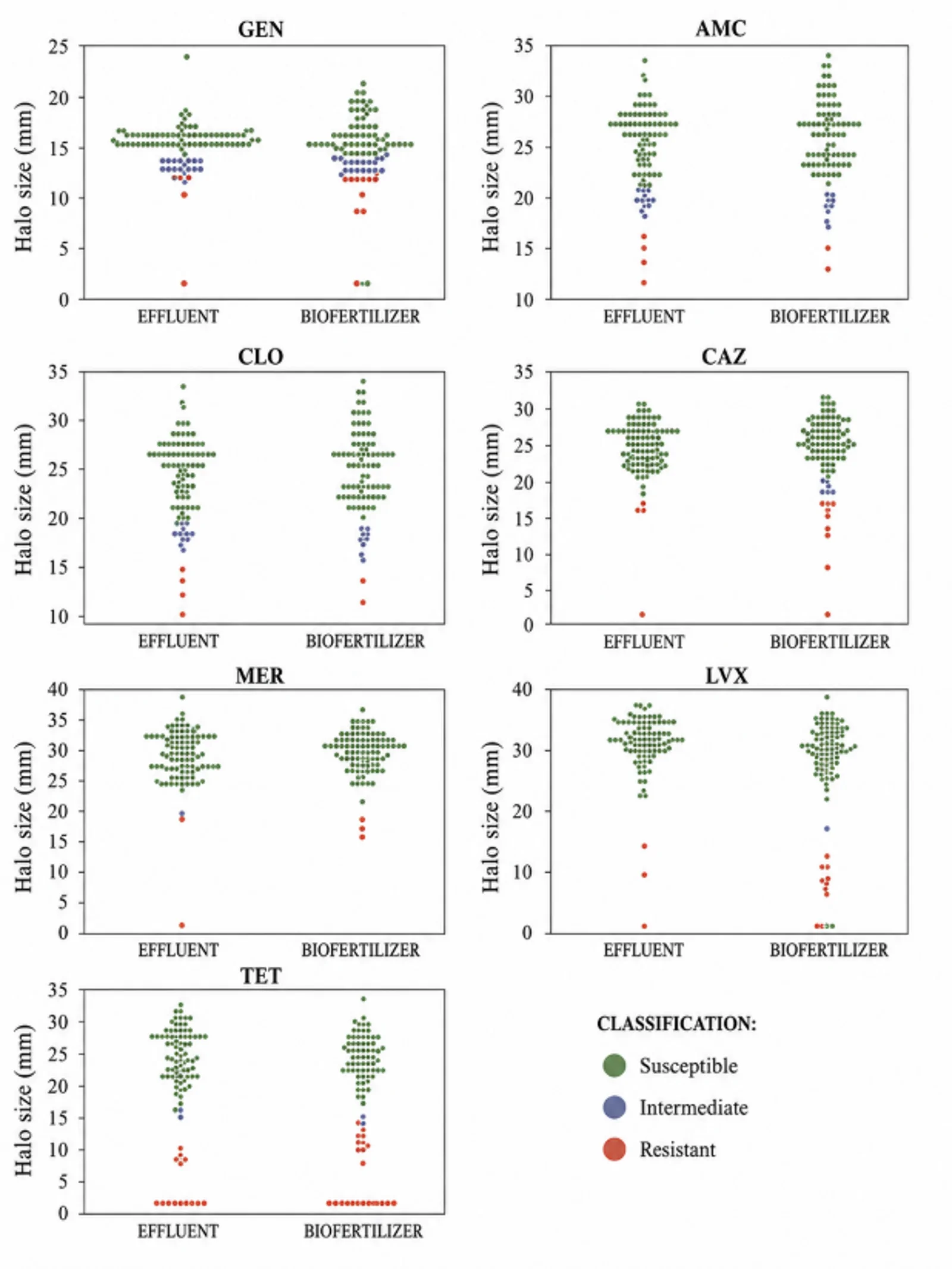
Antimicrobial susceptibility profiles of *E. coli*-like enterobacterial isolates from anaerobic digester influent and biofertilizer. Legend: GEN (Gentamicin), AMC (Amoxicillin-clavulanate), CLO (Chloramphenicol), CAZ (Ceftazidime), MER (Meropenem), LVX (Levofloxacin), TET (Tetracycline). Colors represent CLSI susceptible, intermediate, and resistant classifications

The antimicrobial susceptibility profiles of isolates recovered from effluents and biofertilizers are shown in Fig. 3. Each dot represents the inhibition zone diameter (mm) obtained in the disk diffusion assay for a given antimicrobial agent. According to the breakpoints established, isolates were classified as sensitive (green), intermediate (blue), or resistant (red).

Among the 173 isolates, tetracycline showed the highest frequency of resistance (34/173, 19.7%), followed by gentamicin (18/173, 10.4%), levofloxacin (12/173, 6.9%), amoxicillin-clavulanate (11/173, 6.4%), ceftazidime (11/173, 6.4%), chloramphenicol (7/173, 4.0%), and meropenem (4/173, 2.3%) (Fig. 3). No significant differences in individual antimicrobial resistance frequencies were observed between matrices after multiple-testing correction (Supplementary Table S4).

**Fig. 4:**
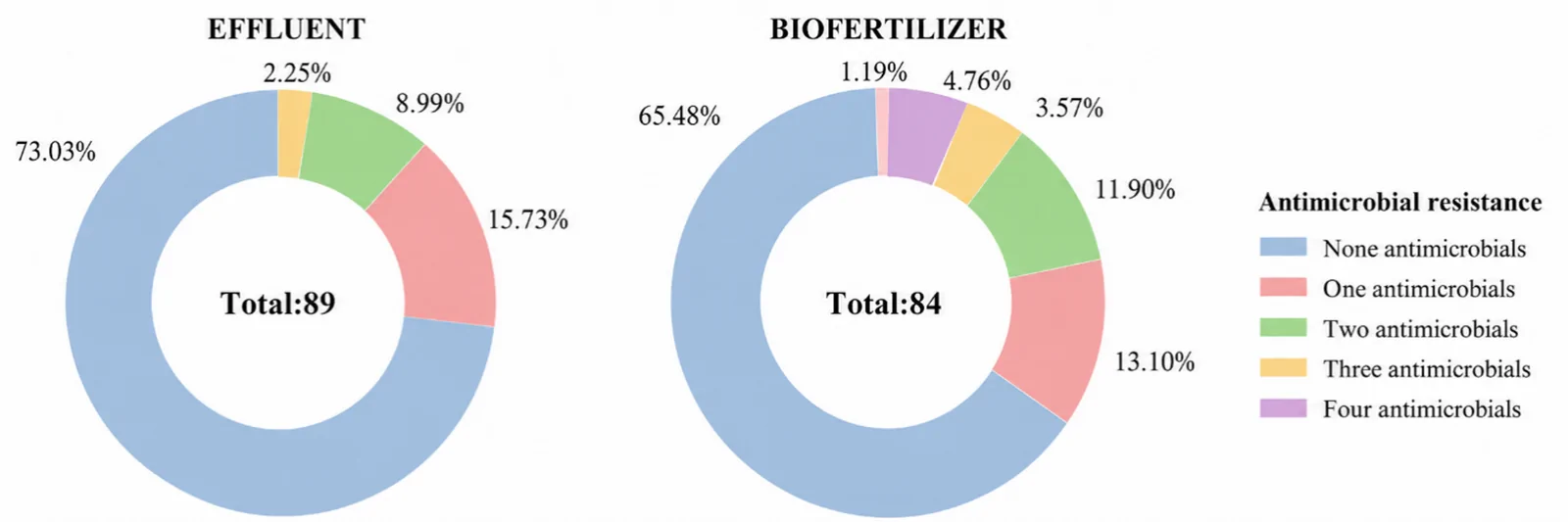
Distribution of the number of tested antimicrobial agents classified as resistant (R) per presumptive *E. coli*-like enterobacterial isolate from effluent and biofertilizer

Overall, phenotypic resistance to at least one of the tested antimicrobials was observed in 30.6% (53/173) of the isolates, comprising 27.0% (24/89) of effluent isolates and 34.5% (29/84) of biofertilizer isolates, with no significant difference between matrices (Fisher’s exact test, *p* = 0.324) (Fig. 4). Among effluent isolates, 14 were resistant to one antimicrobial, eight to two antimicrobials, and two exhibited phenotypic MDR. Among biofertilizer isolates, 11 were resistant to one antimicrobial, 10 to two antimicrobials, and eight exhibited MDR. Resistance to up to five antimicrobials was observed exclusively among biofertilizer isolates. Phenotypic MDR was numerically more frequent in biofertilizer (9.5%, 8/84) than in effluent isolates (2.2%, 2/89), although the difference did not reach statistical significance (Fisher’s exact test, *p* = 0.0521).

Table 1 summarizes the phenotypic MDR patterns identified among the isolates. Two MDR isolates were detected in the effluent, exhibiting the AMC-GEN-TET and AMC-CLO-TET resistance profiles (1.1% each). Among biofertilizer isolates, the most frequent MDR patterns were GEN-TET-LVX and CLO-GEN-TET-CAZ, each detected in two isolates (2.4%). Additional profiles included AMC-GEN-LVX-CAZ, AMC-CLO-TET-LVX, AMC-GEN-MER, and AMC-CLO-GEN-TET-CAZ, each detected in one isolate (1.2%). Overall, MDR phenotypes were heterogeneous, involving resistance to three to five of the tested antimicrobials.

**Table 1:** Phenotypic multidrug-resistance patterns among presumptive *E. coli*-like enterobacterial isolates from anaerobic digester effluent and biofertilizer

Isolates	Resistance phenotype					*n*	%		
Effluent (*n* = 89)	AMC	GEN	TET			1		1.12	
	AMC	CLO	TET			1		1.12	
Biofertilizer (*n* = 84)	CLO	GEN	TET	CAZ		2		2.38	
	GEN	TET	LVX			2		2.38	
	AMC	GEN	MER			1		1.19	
	AMC	CLO	TET	LVX		1		1.19	
	AMC	GEN	LVX	CAZ		1		1.19	
	AMC	CLO	GEN	TET	CAZ	1		1.19	

*AMC* (Amoxicillin + Clavulanic Acid), *CLO* (Chloramphenicol), *GEN* (Gentamicin), *TET* (Tetracycline), *MER* (Meropenem), *LVX* (Levofloxacin), *CAZ* (Ceftazidime)

### Biocide inhibition-zone profiles

**Fig. 5:**
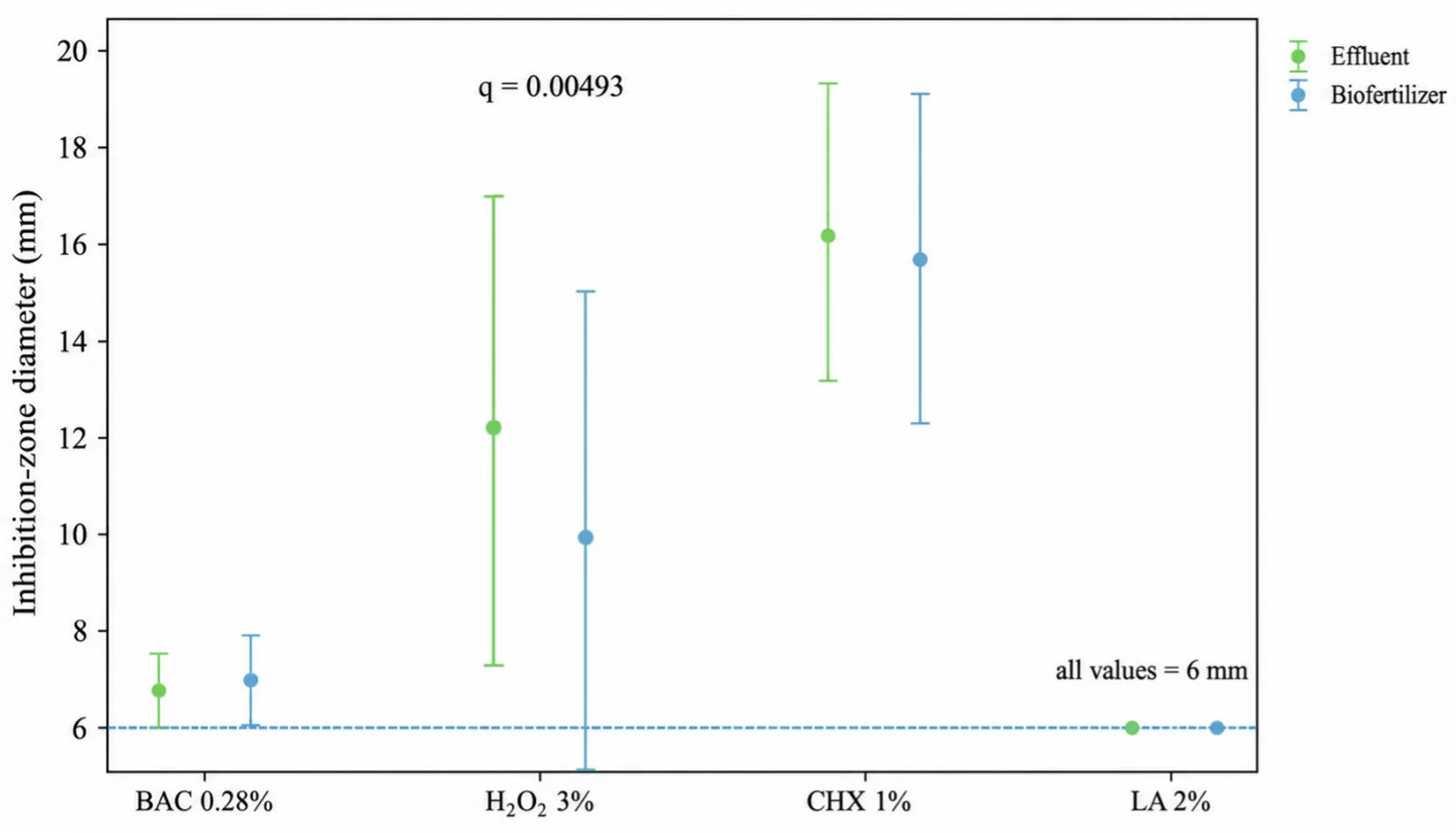
Biocide inhibition-zone profiles of *E. coli*-like enterobacterial isolates from effluent and biofertilizer. Data are presented as mean ± SD. The dashed line indicates the 6-mm disk diameter. H_2_O_2_ inhibition zones differed significantly between matrices after Benjamini–Hochberg correction (q = 0.00493). LA 2% (2% Lactic Acid); BAC 0.28% (0.28% Benzalkonium chloride), H_2_O_2_ 3% (3% hydrogen peroxide); CHX 1% (1% chlorhexidine digluconate)

Biocide inhibition-zone profiles are shown in Fig. 5. The reference strain *E. coli* ATCC 25,922 exhibited inhibition zones of 8 mm for BAC (0.28%), 8 mm for H₂O₂ (3%), 12 mm for CHX (1%), and 6 mm for LA (2%).

Mean inhibition-zone diameters for effluent and biofertilizer isolates were 6.66 ± 0.70 and 6.86 ± 0.93 mm for BAC, 12.03 ± 4.85 and 9.67 ± 5.14 mm for H₂O₂, and 16.10 ± 3.10 and 15.58 ± 3.44 mm for CHX, respectively. H₂O₂ inhibition zones were significantly smaller among biofertilizer isolates (*p* = 0.00164; q = 0.00493), whereas no significant differences were observed for BAC (*p* = 0.247; q = 0.370) or CHX (*p* = 0.533; q = 0.533) (Supplementary Table S5).

An inhibition-zone diameter of 6 mm, corresponding to absence of measurable inhibition beyond the disk diameter, was observed for BAC in 47.2% (42/89) of effluent and 45.2% (38/84) of biofertilizer isolates. For H₂O₂, this profile was observed in 20.2% (18/89) and 47.6% (40/84) of effluent and biofertilizer isolates, respectively, whereas for CHX it was observed in none of the effluent isolates and in 4.8% (4/84) of biofertilizer isolates. All isolates, as well as the ATCC 25,922 reference strain, showed inhibition-zone diameters of 6 mm for LA.

### Genotype–phenotype associations of antimicrobial and biocide resistance

Table 2 summarizes the antimicrobial resistance and biocide inhibition-zone findings for the 28 isolates carrying three or more screened ARGs, including 14 isolates from the effluent and 14 from the biofertilizer. Phenotypic resistance to at least one antimicrobial was observed in 57.1% (8/14) of effluent isolates and 35.7% (5/14) of biofertilizer isolates. The resistant phenotypes included tetracycline, gentamicin, chloramphenicol, amoxicillin-clavulanate, and levofloxacin.

**Table 2:** Isolates carrying three or more screened ARGs and corresponding phenotypic antimicrobial resistance and biocide 6-mm inhibition-zone findings in effluent (*n* = 14) and biofertilizer (*n* = 14). A 6-mm biocide value denotes absence of a measurable inhibition zone beyond the disk diameter

Isolate ID	ARGs	Phenotypic antimicrobial resistance	Biocides with 6-mm zone
Effluent			
2	*ampC*,* bla_TEM*,* qacE∆1*	None	LA
10	*ampC*,* aacA-aphD*,* qacE∆1*	None	H₂O₂, LA
23	*ampC*,* bla_TEM*,* qacE∆1*	None	LA
34	*ampC*,* aacA-aphD*,* qacE∆1*	None	LA
35	*ampC*,* bla_TEM*,* qacE∆1*	None	LA
53	*ampC*,* bla_TEM*,* qacE∆1*	TET	H₂O₂, LA
147	*bla_TEM*,* aacA-aphD*,* qacE∆1*	CLO	LA
158	*ampC*,* bla_TEM*,* qacE∆1*	GEN	BAC, H₂O₂, LA
162	*ampC*,* aacA-aphD*,* qacE∆1*	None	BAC, LA
172	*ampC*,* floR*,* qacE∆1*	GEN, TET	BAC, LA
206	*tetA*,* bla_TEM*,* qacE∆1*	AMC, TET	BAC, H₂O₂, LA
220	*tetA*,* sul1*,*bla_TEM*,* qacE∆1*	AMC, CLO, TET	BAC, LA
230	*tetA*,* bla_TEM*,* qacE∆1*	AMC, TET	BAC, H₂O₂, LA
235	*ampC*,* bla_TEM*,* aacA-aphD*,* qacE∆1*	TET	BAC, LA
Biofertilizer			
2	*ampC*,* aacA-aphD*,* qacE∆1*	None	BAC, H₂O₂, LA
3	*ampC*,* floR*,* qacE∆1*	None	LA
4	*ampC*,* aacA-aphD*,* qacE∆1*	None	LA
5	*floR*,* aacA-aphD*,* qacE∆1*	None	LA
35	*ampC*,* bla_TEM*,* qacE∆1*	None	BAC, LA
62	*ampC*,* aacA-aphD*,* qacE∆1*	None	H₂O₂, LA
130	*ampC*,* bla_TEM*,* qacE∆1*	None	H₂O₂, LA
131	*ampC*,* bla_TEM*,* aacA-aphD*	TET	BAC, H₂O₂, LA
153	*ampC*,* floR*,* aacA-aphD*	None	H₂O₂, LA
209	*tetA*,* floR*,* bla_TEM*,* qacE∆1*	GEN, TET, LVX	BAC, H₂O₂, LA
215	*tetA*,* floR*,* ampC*	None	BAC, H₂O₂, LA
217	*tetA*,* ampC*,* bla_TEM*,* qacE∆1*	AMC	BAC, H₂O₂, LA
266	*tetA*,* bla_TEM*,* qacE∆1*	TET, LVX	LA
306	*tetA*,* ampC*,* qacE∆1*	AMC, CLO, TET, LVX	LA

*ARGs* = Antimicrobial resistance genes, *AMC* (Amoxicillin + Clavulanic Acid), *CLO* (Chloramphenicol), *GEN* (Gentamicin), *TET* (Tetracycline), *LVX* (Levofloxacin)

*BAC* = Benzalkonium chloride, *H*_2_O_2_ = Hydrogen peroxide, *CHX* = Chlorhexidine, *LA* = Lactic acid

Across all 173 isolates, *tet(a)* carriage was strongly associated with tetracycline resistance: 13 of the 17 *tet(a)*-positive isolates exhibited phenotypic resistance (OR = 20.9; Fisher’s exact *p* = 1.04 × 10^− 7^; q = 6.24 × 10^− 7^; Cohen’s κ = 0.436). No significant associations were observed between *ampC* and/or *bla_TEM* and AMC or CAZ resistance, *aacA-aphD* and GEN resistance, *floR* and CLO resistance, or *qacE∆1* and a 6-mm BAC inhibition-zone outcome after multiple-testing correction (Supplementary Table S6).

Biocide inhibition-zone findings for these 28 isolates are also presented in Table 2. All yielded 6-mm measurements for LA, while 6-mm values for BAC and H2O2 occurred variably and were less frequent for CHX. The complete isolate-level dataset is provided in Supplementary Table S7.

## Discussion

The identification and characterization of enterobacteria in agricultural environments, particularly in anaerobic digestion systems, are essential for understanding the dissemination of pathogens and antimicrobial resistance. Cattle and their environment can constitute significant reservoirs of resistant bacteria due to the extensive and continuous use of antimicrobials, which contributes to the selection and persistence of resistant strains (Kimera et al. 2021). In this context, *E. coli* emerges as a key microorganism to monitor, given its relevance as an indicator of fecal contamination and its remarkable ability to acquire antimicrobial resistance.

Although dairy cattle may act as reservoirs of pathogenic *E. coli*, representing a potential public health risk, none of the 173 isolates analyzed in this study carried virulence genes typical of diarrheagenic pathotypes (EPEC, STEC, ETEC, EAEC, and EIEC). This finding does not eliminate potential health risks but indicates that the investigated diarrheagenic markers were not detected among the recovered isolates.

Despite the reassuring absence of virulence genes, antimicrobial resistance remains a substantial concern. Molecular analyses revealed a high prevalence of isolates harboring at least one antimicrobial resistance gene (ARG), with 91.0% of effluent-derived and 83.3% of biofertilizer-derived isolates carrying at least one screened ARG (Figs. 1 and 2). Fourteen isolates from each matrix carried three or more screened ARGs (15.7% of effluent and 16.7% of biofertilizer isolates). These findings are consistent with previous reports emphasizing agricultural environments as hotspots for ARG dissemination (Furlan et al. 2020). The persistence of MDR isolates post-digestion underscores that the process, while reducing total microbial load, may not eliminate resistant subpopulations, with operational factors such as biomass load, hydraulic retention time, and digester conditions influencing the survival of ARGs in digesters (Menezes et al. 2024).

Among the ARGs detected, *qacEΔ1*, a marker associated with reduced susceptibility to quaternary ammonium compounds, was the most frequent, present in 57.8% of isolates. This result is in line with Mwenifumbo et al. (2023), who detected *qacEΔ1* in 59.2% of *E. coli* from dairy calves. In the present study, *qacEΔ1* was significantly more frequent in effluent (70.8%) than in biofertilizer isolates (44.0%; q = 0.00291). β-lactamase genes *ampC* (35.8%) and *bla_TEM* (18.5%) were also highly represented, raising concern due to their plasmid-mediated potential for horizontal transfer (Rodríguez et al. 2020). The prevalence of *bla_TEM* likely reflects local antimicrobial usage practices (Benites et al. 2021), with regional differences emphasizing the influence of environmental and management factors (Chaudhary et al. 2023).

The detection of *tet(a)*, though lower (9.8%) than values reported in some studies (Anderson et al. 2023), was particularly relevant when considered together with the phenotypic results. Tetracycline resistance was observed in 19.7% (34/173) of isolates, corresponding to 15.7% (14/89) of effluent and 23.8% (20/84) of biofertilizer isolates, and *tet(a)* carriage was strongly associated with tetracycline resistance (OR = 20.9; q < 0.001; κ = 0.436). The higher frequency of phenotypic tetracycline resistance than *tet(a)* detection suggests the involvement of other untested tet genes or additional mechanisms such as efflux pumps (Roberts 2005; Gharajalar and Sofiani 2017). Several studies have reported high rates of phenotypic tetracycline resistance in *E. coli* from dairy systems. Uyama et al. (2024) found tetracycline to be the least effective antimicrobial against calf enterobacteria in Canada, while Shoaib et al. (2023), Massé et al. (2023), and Custódio et al. (2024) reported resistance rates ranging from 29% to 80% in isolates from dairy farms in China and North America. This widespread resistance is likely driven by the extensive use of tetracycline, which is excreted largely unmetabolized, maintaining strong environmental selective pressure (Borghi and Palma 2014).

For aminoglycosides, the *aacA-aphD* gene was detected in 19.1% of isolates, whereas phenotypic gentamicin resistance was observed in 10.4% overall (10.1% in effluent and 10.7% in biofertilizer). No significant association was observed between *aacA-aphD* carriage and gentamicin resistance after multiple-testing correction. This discordance indicates that the presence of a resistance-associated marker does not necessarily correspond to the phenotype expressed under the test conditions and may also reflect resistance mechanisms not included in the PCR panel (Scaria et al. 2010; Yang et al. 2024).

Similarly, the detection of *floR* (6.3%), was numerically more frequent in biofertilizer isolates (8.3%) than in effluent isolates (4.5%). Phenotypic chloramphenicol resistance occurred in 2.2% of effluent and 6.0% of biofertilizer isolates, but *floR* carriage was not significantly associated with chloramphenicol resistance. These observations suggest that anaerobic digestion may favor the persistence of specific resistance markers, as reported in other livestock systems (Gelalcha et al. 2023; Custódio et al. 2024).

The *sul1* gene, conferring sulfonamide resistance, was detected at very low prevalence (1.1%, 1/89) in effluent isolates and was absent in biofertilizer samples. This marker was therefore uncommon in the recovered isolate collection.

Phenotypic susceptibility to meropenem remained high across both matrices, with resistance detected in 1.1% of effluent and 3.6% of biofertilizer isolates, aligning with other studies in dairy systems (Gelalcha et al. 2023; Tello et al. 2020). Levofloxacin resistance increased from 3.4% in effluent to 10.7% in biofertilizer, although this difference was not significant after multiple-testing correction. These data are consistent with prior reports in anaerobic digester systems (Naback et al. 2022; Jia et al. 2022). Phenotypic resistance to amoxicillin-clavulanic acid was lower (4.5% in effluent, 8.3% in biofertilizer) than the prevalence of *bla_TEM* (19.1% in effluent, 17.8% in biofertilizer), suggesting incomplete gene expression or regulatory modulation. A similar genotype–phenotype discrepancy was observed for ceftazidime, with resistance increasing from 3.4% in effluent to 9.5% in biofertilizer, despite high prevalence of *ampC* and *bla_TEM* genes. Such divergences can result from gene regulation, environmental conditions, or point mutations affecting phenotypic expression (Yang et al. 2024; Scaria et al. 2010).

When placed in perspective with our previous work in the same biodigester (Menezes et al. 2024), the present findings reveal a scientific progression from phenotypic characterization to a deeper genotypic understanding of resistance. The detection of genes such as *ampC*, *bla-TEM*, and *qacEΔ1* not only complements earlier evidence of multidrug resistance and biofilm formation but also reflects an evolutionary advancement in the knowledge of how anaerobic digestion influences the persistence and diversification of resistance determinants.

Phenotypic MDR was observed in 2.2% (2/89) of effluent and 9.5% (8/84) of biofertilizer isolates. Although numerically higher in biofertilizer, this difference did not reach statistical significance (*p* = 0.0521). MDR profiles were heterogeneous and involved resistance to three to five of the tested antimicrobials. Such findings underscore the risks associated with biofertilizer application in agricultural soils, warranting careful monitoring and management of AMR dissemination (Resende et al. 2014b).

Biocide susceptibility analysis revealed reduced efficacy against common disinfectants used in dairy farming.

Decreased susceptibility of enterobacteria to these biocides can result from multiple mechanisms, including alterations in the cell membrane, production of enzymes that neutralize bactericidal or bacteriostatic effects, and mutations in bacterial DNA (Jones; Joshi, 2021). However, in environmental systems such as anaerobic digesters, reduced biocide efficacy may not be attributable solely to intrinsic bacterial mechanisms. Matrix-related factors must also be considered. High concentrations of organic matter, suspended solids, and dissolved nutrients present in influent and digestate can reduce the effective activity of disinfectants through chemical neutralization, adsorption phenomena, or physical shielding of bacterial cells. Such matrix interference may partially explain reduced apparent susceptibility observed in complex environmental matrices compared to controlled laboratory assays (Geraldes et al. 2021).

BAC widely applied for surface and equipment disinfection in dairy facilities, produced 6-mm inhibition-zone values in 47.2% of effluent and 45.2% of biofertilizer isolates. *qacEΔ1* was detected in 70.8% and 44.0% of these matrices, respectively, and no significant association was observed between *qacEΔ1* carriage and a 6-mm BAC outcome. This genotype–phenotype discrepancy suggests that gene expression may be influenced by regulatory, epigenetic, or environmental factors (Abbood et al. 2023; Leinyuy et al. 2023; Farahani et al. 2024).

Hydrogen peroxide, commonly used for surface sanitization and teat antisepsis before milking, showed significantly smaller inhibition zones among biofertilizer isolates (9.67 ± 5.14 mm) than effluent isolates (12.03 ± 4.85 mm; q = 0.00493), and 6-mm values occurred in 47.6% and 20.2% of isolates, respectively. In contrast, chlorhexidine, widely employed for teat antisepsis and wound treatment in cattle, showed no significant difference between matrices; 6-mm measurements were absent among effluent isolates and occurred in 4.8% of biofertilizer isolates. All isolates and the *E. coli* ATCC 25,922 reference strain yielded 6-mm measurements with lactic acid, indicating that the tested LA condition was non-discriminatory.

Another important aspect revealed by this study is the mismatch between genotypic and phenotypic data, particularly when assessing isolates carrying three or more ARGs (Table 2). While the presence of multiple resistance determinants suggests a strong potential for multidrug resistance, phenotypic resistance was confirmed in only a subset of these isolates. This discrepancy underscores the complexity of resistance expression, as the detection of resistance genes does not necessarily imply their transcriptional activation or phenotypic manifestation (Leinyuy et al. 2023; Farahani et al. 2024). The formal association analysis reinforced this pattern: *tet(a)* was strongly associated with tetracycline resistance, whereas the other evaluated gene–phenotype pairs were not statistically significant after correction. Phenotypic resistance may be influenced by regulatory mechanisms, efflux pump overexpression, stress adaptation responses, or chromosomal mutations not targeted in the molecular screening. In anaerobic environments, sublethal physicochemical stressors may further modulate gene expression and activate global stress responses, potentially altering resistance phenotypes without proportional changes in gene prevalence (Sun et al. 2025). Such discordance highlights the importance of integrating molecular and phenotypic approaches in resistance surveillance.

Although several isolates carrying multiple ARGs also showed 6-mm BAC or H_2_O_2_ values, these co-occurrences were not sufficient to establish cross-resistance. Nevertheless, the combined occurrence of antimicrobial resistance and reduced biocide inhibition warrants monitoring, given the co-selection mechanisms described in other systems (Pereira et al. 2021; Wales and Davies 2015).

The recovery of antimicrobial-resistant isolates from biofertilizer indicates that anaerobic digestion did not eliminate all culturable resistant enterobacteria. This finding raises questions about the suitability of applying untreated or minimally treated biofertilizers to agricultural soils, as such practices could facilitate the spread of MDR bacteria into the food production chain (Xu et al. 2019; Resende et al. 2014a; Aziz et al. 2022).

The operational conditions recorded during sampling provide relevant context for these microbiological findings (Supplementary Table S1). The system operated at ambient temperatures averaging 24.5 °C in March and 18.5 °C in June, with near-neutral pH in effluent and biofertilizer, while total solids decreased from 3.75 to 1.85 g/L in March and from 2.90 to 1.09 g/L in June. In addition, treated liquid from the final biofertilizer pond is reused for floor washing and returns to the treatment train with newly generated cattle wastewater. This recirculation may contribute to bacterial carry-over within the system and may attenuate differences observed between effluent and biofertilizer.

From a One Health perspective, the convergence of antimicrobial resistance genes, multidrug resistance phenotypes, and variable biocide inhibition profiles in enterobacteria isolated from effluent and biofertilizer emphasizes the interconnected risks between animal production, environmental contamination, and human health. The application of biofertilizers containing MDR isolates to croplands could create a critical interface where resistant bacteria are introduced into soil, water, and crop-associated environments (Pandey et al. 2024). This scenario not only threatens animal and public health but also compromises the effectiveness of antimicrobials and biocides that remain essential tools in clinical and agricultural settings.

In this context, it is essential to strengthen waste treatment protocols to ensure more effective microbial inactivation, to promote stricter regulation on the use of antimicrobials and biocides in livestock production, and to expand surveillance strategies that encompass both genotypic and phenotypic assessments of resistance. Future studies should investigate mobile genetic elements and horizontal gene transfer to determine the mobility of the ARGs detected in biofertilizer-associated bacteria.

Beyond its microbiological relevance, this research aligns with broader sustainability goals. By documenting antimicrobial resistance among culturable bacteria recovered from livestock waste treatment systems, it directly contributes to the achievement of the United Nations Sustainable Development Goals (SDGs). Specifically, it supports SDG 12 (“Responsible consumption and production”) by fostering safer and more efficient resource use, and SDG 3 (“Good health and well-being”) by addressing the risks of resistance dissemination under a One Health framework. These connections highlight the societal impact of continuous monitoring and the development of strategies to control resistance in agricultural environments.

## Conclusion

This study demonstrates that culturable enterobacterial isolates carrying antimicrobial resistance genes and phenotypic resistance, including MDR isolates, were recovered from both effluent and biofertilizer in a full-scale dairy anaerobic digestion system. *qacEΔ1* was significantly less frequent among biofertilizer isolates than effluent isolates, while the other screened ARGs did not differ significantly between matrices. Phenotypic MDR was numerically more frequent in biofertilizer but did not reach statistical significance, and H_2_O_2_ inhibition zones were significantly smaller among biofertilizer isolates. The recovery of resistant enterobacteria after treatment highlights the importance of monitoring the microbiological safety of biofertilizer used in agriculture.

These findings underscore the need for integrated management strategies that combine prudent antimicrobial and biocide use, enhanced waste treatment, and comprehensive resistance surveillance. From a One Health perspective, reducing the environmental release of resistant bacteria associated with biofertilizer is essential to protect human, animal, and environmental health, while contributing to broader sustainability goals in agriculture.

## Supplementary Information

Below is the link to the electronic supplementary material.

Supplementary Material 1 (PDF 500 KB)

## Data Availability

The data that supports the findings of this study are available in the supplementary material of this article.

## References

[CR1] Abbood HM, Hijazi K, Gould IM (2023) Chlorhexidine Resistance Or Cross-Resistance, That Is The Question. Antibiotics, V. 12, N. 5, P. 798. 10.3390/Antibiotics12050798 PMC10215778 37237701

[CR2] Ahmed SK, Hussein S, Qurbani K, Ibrahim RH, Fareeq A, Mahmood KA, Mohamed MG (2024) Antimicrobial Resistance: Impacts, Challenges, And Future Prospects. J Med Surg Public Health 2:1–9. 10.1016/J.Glmedi.2024.100081

[CR3] Alvares CA, Stape JL, Sentelhas PC, Gonçalves JL, De M, Sparovek G (2013) Köppen’s Climate Classification Map For Brazil. Meteorologische Zeitschrift, V. 22, N. 6. 711–728. 10.1127/0941-2948/2013/0507

[CR4] Anderson REV, Chalmers G, Murray R, Mataseje L, Pearl DL, Mulvey M, Topp E, Boerlin P (2023) Characterization Of *Escherichia Coli* And Other Enterobacterales Resistant To Extended-Spectrum Cephalosporins Isolated From Dairy Manure In Ontario, Canada. Applied And Environmental Microbiology. Vol. 89, N. 2, P. 1–18. 10.1128/Aem.01869-22 PMC9972979 36695602

[CR5] Aranda KRS, Fagundes-Neto U, Scaletsky ICA (2004) Evaluation Of Multiplex Pcrs For Diagnosis Of Infection With Diarrheagenic *Escherichia Coli* And *Shigella* Spp. J Clin Microbiol V 42:5849–5853. 10.1128/Jcm.42.12.5849-5853.2004 PMC535216 15583323

[CR6] Aziz A, Sengar A, Basheer F, Farooqi IH, Isha MH (2022) Anaerobic Digestion In The Elimination Of Antibiotics And Antibiotic-Resistant Genes From The Environment–A Comprehensive Review. J Environ Chem Eng V 10(1):106423. 10.1016/J.Jece.2021.106423

[CR7] Benites NR, Hora AS, Mello PL, Laes MA, Brandão PE, Melville PA, Zuniga E (2021) Genotypic Evaluation Of Antimicrobial Resistance In *Staphylococcus* Spp. Isolated From Bovine Clinical Mastitis. Arquivo Brasileiro De Medicina Veterinária E Zootecnia, V. 73, N. 2, P. 302–310. 10.1590/1678-4162-11984

[CR8] Bird P, Bastin B, Klass N, Crowley E, Agin J, Goins D, Bakken H, Lingle C, Schumacher A (2020) Evaluation Of The 3m™ Petrifilm™ Rapid *E. Coli*/Coliform Count Plate For The Enumeration Of *E. Coli* And Coliforms: Collaborative Study, First Action: 2018.13. Journal Of Aoac International, V. 103, N. 2. 513–522. 10.1093/Jaocint/Qsz013 33241279

[CR9] Borghi AA, Palma MSA (2014) Tetracycline: production, waste treatment and environmental impact assessment. Brazilian J Pharm Sci 50:25–40. 10.1590/S1984-82502011000100003

[CR10] Burch TR, Firnstahl AD, Spencer SK, Larson RA, Borchardt MA (2022) J Environ Qual V 51:352–363. 10.1002/Jeq2.20350. Fate And Seasonality Of Antimicrobial Resistance Genes During Full-Scale Anaerobic Digestion Of Cattle Manure Across Seven Livestock Production Facilities 35388483

[CR11] Charaya G, Sharma A, Kumar A, Goel P, Singh M (2015) Detection Of Major Mastitis Pathogens By Multiplex Polymerase Chain Reaction Assay In Buffalo Milk. Indian J Anim Sci V 85:122–125. 10.56093/Ijans.V85i2.46562

[CR12] Chaudhary MK, Jadhav I, Banjara MR (2023) Molecular Detection Of Plasmid Mediated *Bla Tem*, *Bla Ctx – M*, And *Bla Shv* Genes In Extended Spectrum β-Lactamase (Esbl) *Escherichia Coli* From Clinical Samples. Annals Of Clinical Microbiology And Antimicrobials, V. 22, N. 1, P. 33. 10.1186/S12941-023-00584-0 PMC10163748 37147617

[CR13] Clsi (2025) Performance Standards For Antimicrobial Susceptibility Testing. 35th Ed. Clsi Supplement M100. Clinical And Laboratory Standards Institute, p 433

[CR14] Custódio DA, Da C, Pereira CR, Gonçalves MS, Costa ACTRB, Oliveira PFR, De; Silva BHP, Da; Carneiro GB, Coura FM, Lage AP, Heinemann MB, Costa GM, Da; Dorneles EMS (2024) Antimicrobial Resistance And Public And Animal Health Risks Associated With Pathogenic *Escherichia Coli* Isolated From Calves. Comparative Immunology, Microbiology And Infectious Diseases. 107:1–8. 10.1016/J.Cimid.2024.102149 38442544

[CR15] Erdaw MM, Contribution (2023) Prospects And Trends Of Livestock Production In Sub-Saharan Africa: A Review. Int J Agricultural Sustain V 21(1):1–16. 10.1080/14735903.2023.2247776

[CR16] Farahani HE, Asgharzadeh S, Golmoradizadeh R, Akrami S, Ghaznavi-Rad E, Mirkalantari S, Masjedian-Jazi F (2024) Resistance To Chlorhexidine And Benzalkonium Chloride In *E. Coli* St131, A. Baumannii, And *P. Aeruginosa* Isolates. Clinical Laboratory, V. 70, N. 1. 10.7754/Clin.Lab.2023.230434 38213201

[CR17] Furlan JPR, Santos LDR, Dos; Ramos MS, Gallo IFL, Stehling EG (2020) Brazilian J Microbiol V 51:1377–1382. 10.1007/S42770-020-00265-6. Fecal Cultivable Aerobic Microbiota Of Dairy Cows And Calves Acting As Reservoir Of Clinically Relevant Antimicrobial Resistance Genes PMC7455631 32246396

[CR18] Gelalcha BD, Gelgie AE, Kerro Dego O (2023) Prevalence And Antimicrobial Resistance Profiles Of Extended-Spectrum Beta-Lactamase-Producing *Escherichia Coli* In East Tennessee Dairy Farms. Front Veterinary Sci 10:1–14. 10.3389/Fvets.2023.1260433 PMC10795760 38239744

[CR19] Geraldes C, Verdial C, Cunha E, Almeida V, Tavares L, Oliveira M, Gil S (2021) Evaluation Of A Biocide Used In The Biological Isolation And Containment Unit Of A Veterinary Teaching Hospital. Antibiot V 10(6):639. 10.3390/Antibiotics10060639 PMC8229411 34071748

[CR20] Gharajalar SN, Sofiani VH (2017) Patterns Of Efflux Pump Genes Among Tetracycline Resistance Uropathogenic *Escherichia Coli* Isolates Obtained From Human Urinary Infections. Jundishapur J Microbiol V 10:1–5. 10.5812/Jjm.40884

[CR21] Jia Y, Mao W, Liu B, Zhang S, Cao J, Xu X (2022) Study On The Drug Resistance And Pathogenicity Of *Escherichia Coli* Isolated From Calf Diarrhea And The Distribution Of Virulence Genes And Antimicrobial Resistance Genes. Front Microbiol V 13:1–10. 10.3389/Fmicb.2022.992111 PMC9815963 36620061

[CR22] Jones IA, Joshi LT, Biocide (2021) Use In The Antimicrobial Era: A Review. Molecules V 26:1–11. 10.3390/Molecules26082276 PMC8071000 33919993

[CR23] Kimera ZI, Mgaya FX, Misinzo G, Mshana SE, Moremi N, Matee MIN (2021) Multidrug-Resistant, Including Extended-Spectrum Beta Lactamase-Producing And Quinolone-Resistant, *Escherichia Coli* Isolated From Poultry And Domestic Pigs In Dar Es Salaam, Tanzania. Antibiotics, V. 10, N. 4, P. 406. 10.3390/Antibiotics10040406 PMC8069735 33918543

[CR24] Leinyuy JF, Ali IM, Ousenu K, Tume CB (2023) Molecular Characterization Of Antimicrobial Resistance Related Genes In *E. Coli*, *Salmonella* And *Klebsiella* Isolates From Broilers In The West Region Of Cameroon. Plos One, V. 18, N. 1, P. E0280150. 10.1371/Journal.Pone.0280150 PMC9833522 36630464

[CR25] Li X, Chen G, Liu L, Wang G (2023) Anaerobic Sludge Digestion Elevates Dissemination Risks Of Bacterial Antibiotic Resistance In Effluent Supernatant. J Environ Manage V 338:1–11. 10.1016/J.Jenvman.2023.117854 37023605

[CR26] Lu Y, Sun R, Zhang C, Ding S, Ying M (2021) In Situ Analysis Of Antibiotic Resistance Genes In Anaerobically Digested Dairy Manure And Its Subsequent Disposal Facilities. Bioresource Technology, V. 333, P. 1–10. 10.1016/J.Biortech.2021.124988 33894444

[CR27] Mapa (MinistérioD, Agricultura, Pecuária E Abastecimento). Mapa Do Leite, Brasília, Df (2023) Available In: Https://Www.Gov.Br/Agricultura/Ptbr/Assuntos/Producao-Animal/Mapadoleite#:~:Text=O%20brasil%20%C3%A9%20o%20terceiro,De%204%20milh%C3%B5es% 20de%20pessoas. Acesso Em: 03 Jan 2024

[CR28] Massé J, Vanier G, Fairbrother JM, De Lagarde M, Arsenault J, Francoz D, Dufour S, Archambault M (2023) Description Of Antimicrobial-Resistant *Escherichia Coli* And Their Dissemination Mechanisms On Dairy Farms. Veterinary Sciences, V. 10, N. 4. 1–18. 10.3390/Vetsci10040242 PMC10144642 37104397

[CR29] Mendonça HV, Ometto JPHB, Otenio MH (2017) Production Of Energy And Biofertilizer From Cattle Wastewater In Farms With Intensive Cattle Breeding. Water, Air, & Soil Pollution, V. 228, P. 1–14. 10.1007/S11270-017-3264-1

[CR30] Menezes KV, Duarte CES, Moreira MG, Moreno TJ, Pereira C, Ucella-Filho VJS, Otenio JGM, Ignacchiti MH, Resende MDC (2024) Enterobacteria In Anaerobic Digestion Of Dairy Cattle Wastewater: Assessing Virulence And Resistance For One Health Security. Water Res 252:121192. 10.1016/J.Watres.2024.121192 38309066

[CR32] Mwenifumbo M, Cookson AL, Zhao S, Fayaz A, Browne AS, Benschop J, Burgess SA (2023) The Characterization Of Antimicrobial Resistant *Escherichia Coli* From Dairy Calves. J Med Microbiol V 72:1–14. 10.1099/Jmm.0.001742 37578342

[CR33] Naback SC, Fernandes ÁJ, Barroso M, Do V, Nascimento CW, Paiva AD, Martins MF, Otenio MH (2022) Identificação E Perfil De Sensibilibiodigestão Anaeróbiade A Antimicrobianos De Bactérias Isolabiodigestão Anaeróbias Na Biodigestão Anaeróbia De Dejetos Suínos E Bovinos. Brazilian Journal Of Development. V.8, N.7. 49091–49108. 10.34117/Bjdv8n7-029

[CR34] Nascimento A, De M, De Paula VR, Dias EHO, Carneiro J, Da C, Otenio MH (2020) Sci Total Environ V 745:1–11. 10.1016/J.Scitotenv.2020.140711. Quantitative Microbial Risk Assessment Of Occupational And Public Risks Associated With Bioaerosols Generated During The Application Of Dairy Cattle Wastewater As Biofertilizer 32763641

[CR36] Pandey S, Doo H, Keum GB, Kim ES, Kwak J, Ryu S, Choi Y, Kang J, Kim S, Lee NR, Oh KK, Lee J-H, Kim HB (2024) Antibiotic Resistance In Livestock, Environment And Humans: One Health Perspective. J Anim Sci Technol 66(2):266. 10.5187/Jast.2023.E129 PMC11016740 38628683

[CR31] Pereira BMP, Wang X, Tagkopoulos I (2021) Biocide-Induced Emergence Of Antibiotic Resistance In *Escherichia Coli*. Frontiers In Microbiology, V. 12, P. 640923. 10.3389/Fmicb.2021.640923 PMC7952520 33717036

[CR37] Pham MN, Nishimura F, Lan JCW, Khoo KS (2024) Recent Advancement Of Eliminating Antibiotic Resistance Bacteria And Antibiotic Resistance Genes In Livestock Waste: A Review. Environ Technol Innov 1–27. 10.1016/J.Eti.2024.103751

[CR38] Ragupathi NKD, Sethuvel DPM, Inbanathan FY, Veeraraghavan B (2018) Accurate Differentiation Of *Escherichia Coli* And *Shigella* Serogroups: Challenges And Strategies. New Microbes And New Infections, V. 21, P. 58–62. 10.1016/J.Nmni.2017.09.003 PMC5711669 29204286

[CR39] Rahut DB, Timsina J, Agriculture-Livestock-Forestry N (2024) In Asia: Potential For Improving Farmers’ Livelihoods And Soil Health, And Adapting To And Mitigating Climate Change. Agricultural Systems, V. 218, P. 1–6. 10.1016/J.Agsy.2024.104012

[CR40] Resende JA, Diniz CG, Silva VL, Otenio MH, Bonnafous A, Arcuri PB, Godon J-J (2014a) Dynamics of antibiotic resistance genes and presence of putative pathogens during ambient temperature anaerobic digestion. J Appl Microbiol 117(6):1689–1699. 10.1111/Jam.12653 25250562

[CR41] Resende JA, Silva VL, Oliveira TLR, Fortunato SO, Carneiro JC, Otenio MH, Diniz CG (2014b) Prevalence and persistence of potentially pathogenic and antibiotic resistant bacteria during anaerobic digestion treatment of cattle manure. Bioresour Technol 153:284–291. 10.1016/J.Biortech.2013.12.007 24374028

[CR42] Roberts MC (2005) Update On Acquired Tetracycline Resistance Genes. Fems Microbiology Letters, V.245, N. 2. 195–203. 10.1016/J.Femsle.2005.02.034 15837373

[CR43] Rodríguez FE, Bolívar-Anillo H, Hoyos Turcios C, Carrillo García L, Serrano Hernández M, Abdellah E (2020) Resistência Antibiótica: El Rol Del Hombre, Los Animales Y El Medio Ambiente Sobre La Salud Humana. Revista Científica Salud Uninorte, V. 36, N. 1. 1–27. 10.14482/Sun.36.1.615

[CR44] Scaria J, Warnick LD, Kaneene JB, May K, Teng C-H, Chang Y-F (2010) Comparison Of Phenotypic And Genotypic Antimicrobial Profiles In *Escherichia Coli* And *Salmonella Enterica* From The Same Dairy Cattle Farms. Molecular And Cellular Probes, V. 24, N. 6, P. 325–345. 10.1016/J.Mcp.2010.07.004 PMC2967584 20688154

[CR45] Shoaib M, He Z, Geng X, Tang M, Hao R, Wang S, Shang R, Wang X, Zhang H, Pu W (2023) The Emergence Of Multi-Drug Resistant And Virulence Gene Carrying *Escherichia Coli* Strains In The Dairy Environment: A Rising Threat To The Environment, Animal, And Public Health. Front Microbiol V 14:1–13. 10.3389/Fmicb.2023.1197579 PMC10382034 37520353

[CR46] Sun H, Levenfors JJ, Brandt C, Schnürer A (2025) Assessing Phenotypic And Genotypic Antibiotic Resistance In Bacillus-Related Bacteria Isolated From Biogas Digestates. Ecotoxicology And Environmental Safety, V. 291, P.117859. 10.1016/J.Ecoenv.2025.117859 39947064

[CR47] Tello M, Ocejo M, Oporto B, Hurtado A (2020) Prevalence Of Cefotaxime-Resistant *Escherichia Coli* Isolates From Healthy Cattle And Sheep In Northern Spain: Phenotypic And Genome-Based Characterization Of Antimicrobial Susceptibility. Applied And Environmental Microbiology, V. 86, N. 15, P. 1–14. 10.1128/Aem.00742-20 PMC7376548 32471914

[CR48] Uyama T, Kelton DF, Morrison EI, De Jong E, Mccubbin KD, Barhema HW, Dufour S, Fonseca M, Mcclure JT, Sanchez J, Heider LC, Renaud DL (2024) Associations Among Antimicrobial Use, Calf Management Practices, And Antimicrobial Resistance In *Escherichia Coli* From A Pooled Fecal Sample In Calves On Canadian Dairy Farms: A Cross-Sectional Study. Journal Of Dairy Science, V. 107, N.7, P. 4961–4972. 10.3168/Jds.2023-24262 38331174

[CR49] Wales AD, Davies RH (2015) Co-Selection Of Resistance To Antibiotics, Biocides And Heavy Metals, And Its Relevance To Foodborne Pathogens. Antibiotics, V. 4, N. 4. 567–604. 10.3390/Antibiotics4040567 PMC4790313 27025641

[CR50] Wallace JS, Garner E, Pruden A, Aga DS (2018) Occurrence And Transformation Of Veterinary Antibiotics And Antibiotic Resistance Genes In Dairy Manure Treated By Advanced Anaerobic Digestion And Conventional Treatment Methods. Environmental Pollution, V. 236, P. 764–772, 10.1016/J.Envpol.2018.02.024 29455089

[CR51] Wang C, Yin X, Xu X, Wang D, Wang Y, Zhang T (2025) Antibiotic Resistance Genes In Anaerobic Digestion: Unresolved Challenges And Potential Solutions. Bioresour Technol 419:132075. 10.1016/J.Biortech.2025.132075 39826759

[CR52] Xu R, Yang Z-H, Zheng Y, Wang Q-P, Bai Y, Liu J-B, Zhang Y-R, Xiong W-P, Lu Y, Fan C-Z (2019) Bioresour Technol 282:179–188. 10.1016/J.Biortech.2019.02.120. Metagenomic Analysis Reveals The Effects Of Long-Term Antibiotic Pressure On Sludge Anaerobic Digestion And Antimicrobial Resistance Risk 30861447

[CR53] Yang B, Xin X, Cao X, Nasifu L, Nie Z, He B (2024) Phenotypic And Genotypic Perspectives On Detection Methods For Bacterial Antimicrobial Resistance In A One Health Context: Research Progress And Prospects. Archives Of Microbiology, V. 206, N. 10, P. 409. 10.1007/S00203-024-04131-Z 39302440

