# Supplementary material for: Phenotypic and genotypic assessment of antimicrobial and biocide resistance in enterobacteria from anaerobic digestion of dairy waste: implications for environmental and public health
Source: World J Microbiol Biotechnol. 2026 Oct 8;42(10):589. doi: 10.1007/s11274-026-05319-9 (PMC13649801; doi:10.1007/s11274-026-05319-9)
Supplement: Supplementary file 1 [file 11274_2026_5319_MOESM1_ESM.pdf]

SUPPLEMENTARY MATERIAL

World Journal of Microbiology and Biotechnology

Phenotypic and genotypic assessment of antimicrobial and biocide resistance in enterobacteria from anaerobic digestion of dairy waste: implications for environmental and public health

Mateus Rodrigues Alves[a], Nicolly Soares Ferreira[b], Max Sulivan Fontes[c], Mariana Fabri Lima[b], Marcelo Henrique Otenio[d], Mariana Drummond Costa Ignacchiti[c], Juliana Alves Resende[a,b,c*]

[a] Graduate Program in Veterinary Sciences, Center for Agricultural Sciences and Engineering - CCAE, Federal University of Espírito Santo - UFES, Alto Universitario, no number, Guararema, Alegre, ES 29500-000, Brazil
[b] Graduate Program in Pharmaceutical Sciences, Center of Health Sciences, Federal University of Espírito Santo (UFES), 29043-900, Vitoria, Espírito Santo State, Brazil.
[c] Department of Pharmacy and Nutrition, Federal University of Espírito Santo - UFES, Alegre, ES 29500-000, Brazil
[d] Embrapa Dairy Cattle - Brazilian Agricultural Research Corporation, Juiz de Fora, Brazil

Corresponding author:
Prof. Dra. Juliana Alves Resende. ORCID iD: http://orcid.org/0000-0002-5476-3754
Mailing address: Federal University of Espírito Santo - UFES, Alto Universitário, no number, 29500-000, Guararema, Alegre - ES, Brazil
E-mail address: juliana.resende@ufes.br

Table S1 - Sampling design and operational/physicochemical characteristics of the full-scale anaerobic digestion system during the field campaign.

Parameter	March 2022	June 2022	Overall/system information
Sampling events	3 paired weekly collections	3 paired weekly collections	12 samples of 500 mL: 6 effluent + 6 biofertilizer
Ambient operating temperature	24.5°C	18.5°C	Operation at ambient temperature
pH (effluent / biofertilizer)	7.07 / 6.96	7.06 / 7.02	Near-neutral pH
Alkalinity, mg $CaCO_3$/L (effluent / biofertilizer)	1448 / 1448	2370 / 2565	
Acidity, mg $CaCO_3$/L (effluent / biofertilizer)	648 / 536	310 / 420	
IA/PA ratio (effluent / biofertilizer)	0.44 / 0.37	0.13 / 0.16	Intermediate alkalinity / partial alkalinity
Total solids, g/L (effluent / biofertilizer)	3.75 / 1.85	2.90 / 1.09	
Working volume			540 m^3
Hydraulic retention time			32 ± 6.4 d in anaerobic digester; ~7 d in final biofertilizer pond
Recirculation			Treated liquid from the final pond was reused for floor washing and returned to the treatment train

IA/PA, intermediate alkalinity/partial alkalinity. VFA, ammonia, organic loading rate, and methane yield were not determined during these sampling events.

Table S2 - Oligonucleotide primers used and fragment sizes for detection of diarrheagenic *E. coli*-associated markers.

Target gene	Sequence (5' – 3')	Amplicon (pb)	Primer concentration	Ta (ºC)	Reference
eae	F: 5′- CTGAACGGCGATTACGCGAA<br>R: 5′- CCAGACGATACGATCCAG	917	5pM	58	Aranda; Fagundes-Neto; Scaletsky, 2004
bfpA	F: 5′- AATGGTGCTTGCGCTTGCTGC<br>R: 5′- GCCGCTTTATCCAAACCTGGTA	326	5pM	58	Aranda; Fagundes-Neto; Scaletsky, 2004
CVD432	F: 5′- CTGGCGAAAGACTGTATCAT<br>R: 5′- CAATGTATAGAAATCCGCTGTT	630	5pM	58	Aranda; Fagundes-Neto; Scaletsky, 2004
LT gene	F: 5′- GGCGACAGATTATACCGTGC<br>R: 5′- CGGTCTCTATATTCCCTGTT	450	5pM	50	Aranda; Fagundes-Neto; Scaletsky, 2004
ST gene	F: 5′- ATTTTTMTTTCTGTATTRTCTT<br>R: 5′- CACCCGGTACARGCAGGATT	190	6,5pM	50	Aranda; Fagundes-Neto; Scaletsky, 2004
ipaH	F: 5′- GTTCCTTGACCGCCTTTCCGATACCGTC<br>R: 5′- GCCGGTCAGCCACCCTCTGAGAGTAC	600	10pM	50	Aranda; Fagundes-Neto; Scaletsky, 2004
stx1	F: 5′- ATAAATCGCCATTCGTTGACTAC<br>R: 5′- AGAACGCCCACTGAGATCATC	180	4pM	50	Aranda; Fagundes-Neto; Scaletsky, 2004
stx2	F: 5′- GGCACTGTCTGAAACTGCTCC<br>R: 5′- TCGCCAGTTATCTGACATTCTG	255	3pM	50	Aranda; Fagundes-Neto; Scaletsky, 2004

Table S3 - Oligonucleotide primers used and fragment sizes for resistance gene identification.

Target gene	Sequence (5' – 3')	Amplicon (pb)	Primer concentration	Ta (ºC)	Reference
bla_TEM	F: 5′-GTG CGC GGA ACC CCT ATT<br>R: 5′-TTA CCA ATG CTT AAT CAG TGA GGC	968	5pM	56	Jones *et al.*, 2009
ampC	F: 5′- ATA ACC ACC CAG TCA CGC<br>R: 5′- CAG TAG CGA GAC TGC GCA	630	5pM	60	Odeh *et al.*, 2002
sul1	F: 5′- ATG GTG ACG GTG TTC GGC ATT CTG A<br>R: 5′- CTA GGC ATG ATC TAA CCC TCG GTC T	815	5pM	64	Grape; Sudstrom; Kronvall, 2003
aacA-aphD	F: 5′- ATC CAA GAG CAA TAA GGG CAT AC<br>R: 5′- GCCACACTATCATAACCACTACC	226	5pM	60	Khan *et al.*, 2011
tet(a)	F: 5′- GCT ACA TCC TGC TTG CCT TC<br>R: 5′- CAT AGA TCG CCG TGA AGA GG	210	6,5pM	55	Ng *et al.*, 2001
floR	F: 5′- GTCATTCCTCACCTTCATCCTAC<br>R: 5′- GACACCAGCACTGCCATTG	243	10pM	60	Khan *et al.*, 2011
qacEΔ1	F: 5'- AGG CTG GCT TTT TCT TGT TAT<br>R: 5'- CCT CCG CAG CGA CGA TTC CAC GAT	328	5pM	60	Khan *et al.*, 2011

Table S4 - Statistical comparisons of screened ARG frequencies and antimicrobial resistance phenotypes between effluent and biofertilizer isolates.

Outcome	Effluent n/N (%)	Biofertilizer n/N (%)	OR*	Exact p	BH q
qacEΔ1	63/89 (70.8)	37/84 (44.0)	3.08	0.00041521	0.00290647
ampC	33/89 (37.1)	29/84 (34.5)	1.12	0.753134	0.988775
aacA-aphD	16/89 (18.0)	17/84 (20.2)	0.864	0.846758	0.988775
bla_TEM	17/89 (19.1)	15/84 (17.9)	1.09	0.847521	0.988775
tet(a)	7/89 (7.9)	10/84 (11.9)	0.632	0.447756	0.988775
floR	4/89 (4.5)	7/84 (8.3)	0.518	0.36055	0.988775
sul1	1/89 (1.1)	0/84 (0.0)	∞	1	1
At least one screened ARG	81/89 (91.0)	70/84 (83.3)	2.02	0.171142	—
≥3 screened ARGs	14/89 (15.7)	14/84 (16.7)	0.933	1	—
AMC resistance	4/89 (4.5)	7/84 (8.3)	0.518	0.36055	0.420641
CLO resistance	2/89 (2.2)	5/84 (6.0)	0.363	0.267034	0.420641
GEN resistance	9/89 (10.1)	9/84 (10.7)	0.938	1	1
TET resistance	14/89 (15.7)	20/84 (23.8)	0.597	0.250581	0.420641
MER resistance	1/89 (1.1)	3/84 (3.6)	0.307	0.356568	0.420641
LVX resistance	3/89 (3.4)	9/84 (10.7)	0.291	0.0742565	0.420641
CAZ resistance	3/89 (3.4)	8/84 (9.5)	0.331	0.1241	0.420641
Resistance to ≥1 antimicrobial	24/89 (27.0)	29/84 (34.5)	0.7	0.323601	—
Phenotypic MDR	2/89 (2.2)	8/84 (9.5)	0.218	0.0521416	—

*OR compares effluent with biofertilizer. BH q values were adjusted separately within the seven individual ARG tests and the seven individual antimicrobial tests. Composite outcomes are shown with unadjusted Fisher exact p-values.

Table S5 - Comparison of biocide inhibition-zone diameters between effluent and biofertilizer isolates.

Compound	Effluent mean ± SD (mm)	Biofertilizer mean ± SD (mm)	Effluent median [IQR]	Biofertilizer median [IQR]	Mann–Whitney p	BH q
BAC	6.66 ± 0.70	6.86 ± 0.93	7.0 [6.0–7.0]	7.0 [6.0–7.5]	0.247	0.370
H_2O_2	12.03 ± 4.85	9.67 ± 5.14	14.0 [7.0–16.0]	7.0 [6.0–14.63]	0.00164	0.00493
CHX	16.10 ± 3.10	15.58 ± 3.44	16.0 [14.0–18.5]	16.0 [14.0–18.0]	0.533	0.533

LA was not tested inferentially because all measurements were 6 mm in both matrices. BH, Benjamini–Hochberg; IQR, interquartile range.

Table S6 - Genotype–phenotype association analyses across all 173 isolates.

Marker	Outcome	G+/R+	G+/R−	G−/R+	G−/R−	OR	Exact p	BH q	κ
ampC and/or bla_TEM	AMC resistance	6	76	5	86	1.36	0.758247	1	0.019
ampC and/or bla_TEM	CAZ resistance	3	79	8	83	0.394	0.218556	0.437112	-0.054
aacA-aphD	GEN resistance	3	30	15	125	0.833	1	1	-0.020
tet(a)	TET resistance	13	4	21	135	20.9	1.03962e-07	6.23772e-07	0.436
floR	CLO resistance	0	11	7	155	0	1	1	-0.052
qacEΔ1	BAC: 6-mm zone	42	58	38	35	0.667	0.21811	0.437112	-0.097

For antimicrobial pairs, R+ denotes the CLSI resistant category and intermediate results were treated as non-resistant in the binary association analysis. For *qacEΔ1*/BAC, the outcome was a 6-mm BAC value (no inhibition beyond the disk). MER and LVX were not paired because corresponding molecular targets were not screened; *sul1* was not paired because no sulfonamide was phenotypically tested. BH adjustment was applied across the six prespecified association tests.

Table S7 - Isolate-level antimicrobial resistance genes (ARGs), antimicrobial susceptibility classifications, and biocide inhibition-zone diameters.

Effluent Isolate ID	ARGs	Biocide inhibition-zone diameter (mm)				Antimicrobial susceptibility profile						
		**BAC**	**H_2O_2**	**CHX**	**LA**	**AMC**	**CLO**	**GEN**	**TET**	**MER**	**LVX**	**CAZ**
**1**	*bla_TEM*; *qacEΔ1*	7.5	12	15	6	S	S	S	S	S	S	S
**2**	*ampC*; *bla_TEM*; *qacEΔ1*	7	14	16	6	S	S	S	S	S	S	S
**7**	*qacEΔ1*	7.5	22.5	19	6	S	S	S	S	S	S	S
**9**	*tet(a); ampC*	7	6	13.5	6	S	S	S	S	S	S	S
**10**	*ampC*; *aacA-aphD*; *qacEΔ1*	7	6	13	6	S	S	S	S	S	S	S
**14**	*bla_TEM*	6	13	15	6	S	S	S	S	S	S	S
**19**	*qacEΔ1*	6	7	13	6	S	S	S	S	S	S	S
**21**	*ampC*	6	17	16	6	S	S	S	S	S	S	S
**23**	*ampC*; *bla_TEM*; *qacEΔ1*	8	17	18.5	6	S	S	S	S	S	S	S
**25**	*ampC*; *qacEΔ1*	7	7	16	6	S	S	S	S	S	S	S
**28**	*qacEΔ1*	6	11	16.5	6	S	S	S	S	S	S	S
**29**	*qacEΔ1*	8	15	16	6	S	S	S	S	S	S	S
**30**	*qacEΔ1*	6	6	11	6	S	S	S	R	S	S	S
**34**	*ampC*; *aacA-aphD*; *qacEΔ1*	7	18.5	17.5	6	S	S	S	S	S	S	S
**35**	*ampC*; *bla_TEM*; *qacEΔ1*	7	7	12	6	S	S	S	S	S	S	S
**38**	*ampC*; *qacEΔ1*	7	7	19	6	S	S	S	S	S	S	S
**39**	*ampC*; *qacEΔ1*	8	6	12	6	S	S	S	S	S	S	S
**51**	no genes detected	6	6	13	6	S	S	S	S	S	S	S
**52**	*ampC*; *qacEΔ1*	6	6	13	6	S	S	S	S	S	S	S
**53**	*ampC*; *bla_TEM*; *qacEΔ1*	7	6	14	6	S	S	S	R	S	S	S
**56**	*ampC*; *qacEΔ1*	7	18	21	6	S	S	S	S	S	S	S
**58**	*qacEΔ1*	6	15	17	6	S	S	S	S	S	S	S
**63**	*ampC*	6	6	16	6	S	S	S	R	S	S	S
**68**	*ampC*	6	7	16	6	S	S	S	S	S	S	S
**74**	*ampC*; *qacEΔ1*	7	7	15	6	S	S	S	S	S	S	S
**80**	*ampC*; *qacEΔ1*	7.25	13.7	15.75	6	S	S	S	S	S	S	S
**84**	*ampC*	7	18	19	6	S	S	S	S	S	S	S

Effluent Isolate ID	ARGs	Biocide inhibition-zone diameter (mm)				Antimicrobial susceptibility profile						
		**BAC**	**H_2O_2**	**CHX**	**LA**	**AMC**	**CLO**	**GEN**	**TET**	**MER**	**LVX**	**CAZ**
**99**	*ampC*; *qacEΔ1*	7	7	13	6	S	S	S	S	S	S	S
**100**	*qacEΔ1*	6	6.5	12	6	S	S	S	S	S	S	S
**102**	*qacEΔ1*	6	8	12	6	S	S	S	S	S	S	S
**108**	no genes detected	6	6	10	6	S	S	R	S	S	S	S
**121**	*ampC*; *qacEΔ1*	6	19	22	6	S	S	S	S	S	S	S
**122**	*ampC*; *qacEΔ1*	6	6	11	6	S	S	S	S	S	S	S
**128**	*ampC*	8	17	19	6	S	S	R	S	S	S	S
**131**	*ampC*	7	16	22	6	I	S	I	S	S	S	S
**133**	*qacEΔ1*	8	7	14	6	S	S	R	S	S	S	S
**134**	*aacA-aphD*	6	7	14	6	S	S	R	S	S	S	R
**139**	*aacA-aphD*; *qacEΔ1*	7	15.5	20	6	S	S	R	R	S	S	S
**147**	*bla_TEM; aacA-aphD; qacEΔ1*	7	15	14	6	S	R	S	S	S	S	S
**151**	*aacA-aphD*; *qacEΔ1*	7	7	12	6	S	S	S	S	S	S	S
**153**	no genes detected	8	8	15	6	S	S	S	S	S	S	S
**156**	*bla_TEM*	7	7	14	6	S	S	S	S	S	S	S
**157**	*aacA-aphD*; *qacEΔ1*	6.5	6.5	13	6	S	S	S	S	S	S	S
**158**	*ampC*; *bla_TEM*; *qacEΔ1*	6	6	13	6	S	S	R	S	S	S	S
**159**	*floR*; *qacEΔ1*	7	6	11	6	R	S	R	R	I	S	S
**161**	*ampC*; *qacEΔ1*	6	16	22	6	S	S	I	S	S	S	S
**162**	*ampC*; *aacA-aphD*; *qacEΔ1*	6	15	20	6	S	S	I	S	S	S	S
**164**	*qacEΔ1*	6	17	19	6	S	S	I	S	S	S	S
**167**	*qacEΔ1*	6	16	17	6	S	S	I	S	S	S	S
**169**	no genes detected	6	18	18	6	S	S	I	S	S	S	S
**171**	*qacEΔ1*	7	19	19	6	S	S	I	S	S	S	S
**172**	*ampC*; *floR*; *qacEΔ1*	6	16	20	6	S	S	R	R	S	S	S
**174**	*floR*	6	11.5	17	6	S	S	S	S	S	S	S
**175**	*ampC*; *qacEΔ1*	7	7	15	6	S	S	S	S	S	S	S
**176**	*qacEΔ1*	6	14	20	6	S	S	S	S	S	S	S
**177**	*qacEΔ1*	7	6	12.5	6	S	S	S	S	S	S	S

Effluent Isolate ID	ARGs	Biocide inhibition-zone diameter (mm)				Antimicrobial susceptibility profile						
		BAC	H_2O_2	CHX	LA	AMC	CLO	GEN	TET	MER	LVX	CAZ
**178**	no genes detected	8	7.5	16	6	S	S	S	S	S	S	S
**180**	*qacEΔ1*	6.5	6	19.5	6	S	S	S	S	R	S	S
**186**	no genes detected	8	6	11.5	6	I	S	S	S	S	S	S
**188**	*qacEΔ1*	8	19	20	6	I	S	S	S	S	S	S
**190**	*qacEΔ1*	6	17.5	17	6	I	S	S	S	S	S	S
**191**	*tet(a)*; *qacEΔ1*	6	14.5	17	6	S	S	S	R	S	S	S
**200**	*qacEΔ1*	7.5	15	18	6	S	S	S	S	S	S	S
**204**	*qacEΔ1*	6	17	21.5	6	I	S	S	S	S	S	S
**206**	*tet(a)*; *bla_TEM*; *qacEΔ1*	6	6	14	6	R	S	S	R	S	S	S
**207**	*qacEΔ1*	6	16	17.5	6	I	S	S	S	S	S	S
**208**	*qacEΔ1*	7.5	17.5	21	6	S	S	S	S	S	S	S
**212**	*qacEΔ1*	8	15.5	18	6	S	S	S	S	S	S	S
**216**	*floR*; *qacEΔ1*	6	20	21	6	S	S	S	R	S	S	S
**220**	*tet(a)*; *sul1*; *bla_TEM*; *qacEΔ1*	6	7	17	6	R	R	S	R	S	S	S
**221**	*aacA-aphD*; *qacEΔ1*	7	15.5	19	6	I	S	S	S	S	S	S
**222**	*aacA-aphD*; *qacEΔ1*	6	15	18	6	S	S	S	S	S	S	S
**223**	no genes detected	6	13	22	6	I	S	S	S	S	S	S
**224**	*ampC*; *qacEΔ1*	7	14	16	6	S	S	S	S	S	S	S
**230**	*tet(a)*; *bla_TEM*; *qacEΔ1*	6	6	10	6	R	S	S	R	S	S	S
**233**	*qacEΔ1*	7	14	16	6	S	S	S	S	S	S	I
**235**	*ampC*; *bla_TEM*; *aacA-aphD*; *qacEΔ1*	6	15.5	16.5	6	I	I	S	R	S	S	S
**237**	*aacA-aphD*	7	15	16	6	S	S	S	S	S	S	R
**250**	*ampC*; *qacEΔ1*	6	14	14	6	S	S	S	S	S	S	S
**272**	no genes detected	6	14.5	15.5	6	S	S	R	S	S	S	S
**273**	*qacEΔ1*	7.5	14	17	6	I	S	S	S	S	S	S
**292**	*ampC*; *bla_TEM*	6	14	15.5	6	S	I	I	S	S	S	S
**299**	*ampC*	7	17	20	6	S	S	S	I	S	R	R
**300**	*bla_TEM*; *qacEΔ1*	7	16	16.5	6	S	S	S	S	S	S	S
**301**	*bla_TEM*; *qacEΔ1*	6	6	11	6	S	S	I	S	S	S	S

Effluent Isolate ID	ARGs	Biocide inhibition-zone diameter (mm)				Antimicrobial susceptibility profile						
		**BAC**	**H_2O_2**	**CHX**	**LA**	**AMC**	**CLO**	**GEN**	**TET**	**MER**	**LVX**	**CAZ**
**302**	*tet(a)*; *aacA-aphD*	7	15	14	6	S	S	I	R	S	R	S
**311**	*tet(a)*; *aacA-aphD*	7	16	16	6	S	S	I	R	S	R	S
**313**	*aacA-aphD*	6	15.5	15	6	I	S	I	I	S	S	S
**319**	*bla_TEM*; *aacA-aphD*	6	15	17	6	I	S	I	S	S	S	S

ARGs: isolates carrying three or more screened ARGs are shown in red for descriptive identification only.

Biocides: BAC (0.28%) - benzalkonium chloride; H_2O_2 (3%) - hydrogen peroxide; CHX (1%) - chlorhexidine; LA (2%) - lactic acid. Values are inhibition-zone diameters (mm). A value of 6 mm indicates no measurable inhibition beyond the disk diameter.

Antimicrobials: AMC (30 µg) - amoxicillin-clavulanic acid; CLO (30 µg) - chloramphenicol; GEN (10 µg) - gentamicin; TET (30 µg) - tetracycline; MER (10 µg) - meropenem; LVX (5 µg) - levofloxacin; CAZ (30 µg) - ceftazidime. S - susceptible; I - intermediate; R - resistant.

Biofertilizer Isolate ID	ARGs	Biocide inhibition-zone diameter (mm)				Antimicrobial susceptibility profile						
		BAC	H_2O_2	CHX	LA	AMC	CLO	GEN	TET	MER	LVX	CAZ
**2**	*ampC*; *aacA-aphD*; *qacEΔ1*	6	6	14.5	6	S	S	S	S	S	S	S
**3**	*ampC*; *floR*; *qacEΔ1*	7	18.5	19	6	S	S	S	S	S	S	S
**4**	*ampC*; *aacA-aphD*; *qacEΔ1*	7	15.5	21	6	S	S	S	S	S	S	S
**5**	*floR*; *aacA-aphD*; *qacEΔ1*	8	19	18	6	S	S	S	S	S	S	S
**7**	*aacA-aphD*	6	6	13	6	S	S	S	R	S	S	S
**8**	*qacEΔ1*	6	6	17	6	S	S	S	S	S	S	S
**15**	*qacEΔ1*	7	6.5	15	6	S	S	S	S	S	S	S
**18**	*qacEΔ1*	7	6	19	6	S	S	S	S	S	S	S
**19**	*qacEΔ1*	6	14.5	16	6	S	S	S	S	S	S	S
**33**	*aacA-aphD*	6	16	22	6	S	S	S	S	S	S	S
**34**	*ampC*	6	6	19	6	S	S	S	S	S	S	S
**35**	*ampC*; *bla_TEM*; *qacEΔ1*	6	7.5	15	6	S	S	S	S	S	S	S
**36**	*ampC*	6	6	15.5	6	S	S	S	S	S	S	S
**37**	*ampC*; *qacEΔ1*	6	6	12	6	S	S	I	S	S	S	S
**39**	*floR*	7	7	19	6	S	S	S	S	S	S	S
**40**	no genes detected	6	6	17	6	S	S	S	S	S	S	S
**48**	no genes detected	8	7	15	6	S	S	S	R	R	S	S
**55**	*ampC*; *qacEΔ1*	6	6	15.5	6	S	S	S	S	S	S	I
**62**	*ampC*; *aacA-aphD*; *qacEΔ1*	7	6	19	6	I	S	S	S	S	S	S
**61**	*ampC*	9	6	13	6	S	S	S	R	S	S	S
**64**	no genes detected	6	16	17	6	S	S	S	S	S	S	I
**67**	*bla_TEM*	6	18	19	6	I	S	S	S	S	S	S
**68**	*ampC*	6	6	11	6	S	S	S	S	S	S	S
**70**	no genes detected	7	7	14	6	S	S	S	S	S	S	S
**71**	no genes detected	6	20	20	6	S	S	S	S	S	S	S
**74**	*qacEΔ1*	7	7	16	6	R	I	I	S	S	S	R
**78**	*ampC*	7.5	6	17	6	S	S	I	S	I	S	S
**79**	no genes detected	7.5	23	22	6	S	S	I	R	S	R	S

Biofertilizer Isolate ID	ARGs	Biocide inhibition-zone diameter (mm)				Antimicrobial susceptibility profile						
		BAC	H_2O_2	CHX	LA	AMC	CLO	GEN	TET	MER	LVX	CAZ
**81**	*ampC*; *qacEΔ1*	6	7	15	6	S	I	S	S	R	I	S
**84**	*qacEΔ1*	7.54	13	16.15	6	S	S	I	S	S	S	S
**87**	no genes detected	6.5	22	21	6	S	S	I	R	S	S	S
**91**	*ampC*; *qacEΔ1*	6.5	19	20.5	6	S	S	R	S	S	S	S
**97**	*ampC*	7.54	13	16.15	6	R	S	R	S	S	R	R
**98**	no genes detected	7.5	6	15	6	I	R	R	R	S	S	R
**114**	*aacA-aphD*; *qacEΔ1*	6	6	15	6	S	R	R	R	S	S	R
**116**	*aacA-aphD*; *qacEΔ1*	6	17	19	6	S	S	S	S	S	S	S
**119**	*aacA-aphD*; *qacEΔ1*	7	17	17	6	S	S	S	S	S	S	S
**128**	*aacA-aphD*	6	6	16	6	S	S	S	S	S	S	S
**121**	*aacA-aphD*	6	6	11	6	S	S	S	S	S	S	S
**125**	*aacA-aphD*	6	6	18.5	6	S	S	S	S	S	S	S
**130**	*ampC; bla_TEM; qacEΔ1*	7	6	16	6	S	S	S	S	S	S	S
**131**	*ampC; bla_TEM; aacA-aphD*	6	6	15.5	6	S	S	S	R	S	S	S
**136**	no genes detected	7	17	17.5	6	S	S	S	S	S	S	S
**132**	*ampC*	8	16	16	6	S	S	S	S	S	S	S
**147**	*aacA-aphD*	6	6	14	6	S	S	S	S	S	S	S
**148**	*ampC*; *aacA-aphD*	6	6	13.5	6	S	S	S	S	S	S	S
**149**	*qacEΔ1*	8.5	6	14	6	S	S	S	S	S	S	S
**153**	*ampC; floR; aacA-aphD*	7	6	19	6	S	S	S	S	S	S	S
**152**	*qacEΔ1*	8	6	12	6	S	S	I	S	S	S	S
**157**	no genes detected	6	6	12	6	S	S	S	S	S	S	S
**186**	no genes detected	9	17.5	21	6	S	S	R	R	S	R	S
**191**	*ampC*	7	18	19	6	S	S	S	S	S	S	S
**193**	no genes detected	6	18.5	19	6	I	R	R	S	S	S	S
**197**	*tet(a)*; *ampC*	6	17	17	6	S	S	S	S	S	S	I
**199**	*tet(a)*	6	6	10	6	S	S	S	R	S	R	S
**201**	*tet(a)*	6	6	19	6	S	S	S	R	S	R	S
**203**	*tet(a)*; *bla_TEM*	6	6	13.5	6	I	S	S	R	S	S	S

Biofertilizer Isolate ID	ARGs	Biocide inhibition-zone diameter (mm)				Antimicrobial susceptibility profile						
		BAC	H_2O_2	CHX	LA	AMC	CLO	GEN	TET	MER	LVX	CAZ
**209**	*tet(a)*; *floR*; *bla_TEM*; *qacEΔ1*	6	6	12	6	S	S	R	R	S	R	S
**215**	*tet(a)*; *ampC*; *floR*	6	6	13	6	S	I	I	S	S	S	S
**217**	*tet(a)*; *ampC*; *bla_TEM*; *qacEΔ1*	6	6	19	6	R	S	S	I	S	S	S
**218**	*bla_TEM*; *qacEΔ1*	8	18	18	6	S	S	S	R	S	S	R
**220**	*floR*	7	6	14	6	S	S	I	S	S	S	S
**221**	no genes detected	6	15	16	6	S	S	I	R	S	S	S
**222**	*bla_TEM*	6	6	10.5	6	S	I	S	S	S	S	I
**228**	*ampC*	8	10	17	6	S	S	S	R	S	S	S
**230**	*bla_TEM*	7	7	15	6	I	S	I	S	S	S	S
**242**	*bla_TEM*	8	9	15	6	I	S	I	S	S	S	S
**245**	*bla_TEM*	9	7.5	6	6	I	S	S	S	S	S	S
**250**	*qacEΔ1*	7	7	14	6	S	S	S	S	S	S	R
**262**	*bla_TEM*; *qacEΔ1*	7	7	12	6	S	S	I	S	S	S	S
**264**	*qacEΔ1*	7	6	15	6	S	I	S	S	S	S	S
**265**	*ampC*; *bla_TEM*	7.5	7	14	6	S	S	S	S	S	S	S
**266**	*tet(a)*; *bla_TEM*; *qacEΔ1*	7.5	17	17	6	S	S	S	R	S	R	S
**277**	*ampC* ; *qacEΔ1*	8.5	6	16	6	S	I	I	S	S	S	S
**280**	*tet(a)*	8	7	6	6	S	S	S	R	S	R	S
**281**	*ampC*	9	8.5	6	6	S	S	S	S	S	S	S
**282**	*qacEΔ1*	6	8	14	6	I	S	S	S	S	S	S
**295**	*aacA-aphD*; *qacEΔ1*	8	7.5	14	6	S	S	I	S	S	S	S
**306**	*tet(a)*; *ampC*; *qacEΔ1*	8	8	15	6	R	R	S	R	S	R	S
**307**	*qacEΔ1*	7.5	6	15	6	R	S	R	I	R	S	S
**309**	*qacEΔ1*	6	6	6	6	S	S	S	S	S	S	S
**310**	no genes detected	8	6	17	6	R	S	S	S	S	S	R
**311**	*qacEΔ1*	8	6	16.5	6	S	S	S	S	S	S	S
**312**	*qacEΔ1*	6	14	17	6	R	R	R	R	S	S	R

ARGs: isolates carrying three or more screened ARGs are shown in red for descriptive identification only.

Biocides: BAC (0.28%) - benzalkonium chloride; H_2O_2 (3%) - hydrogen peroxide; CHX (1%) - chlorhexidine; LA (2%) - lactic acid. Values are inhibition-zone diameters (mm). A value of 6 mm indicates no measurable inhibition beyond the disk diameter.

Antimicrobials: AMC (30 µg) - amoxicillin-clavulanic acid; CLO (30 µg) - chloramphenicol; GEN (10 µg) - gentamicin; TET (30 µg) - tetracycline; MER (10 µg) - meropenem; LVX (5 µg) - levofloxacin; CAZ (30 µg) - ceftazidime. S - susceptible; I - intermediate; R - resistant.

REFERENCES

ARANDA, K. R. S.; FAGUNDES-NETO, U; SCALETSKY, I. C. A. Evaluation of Multiplex PCRs for Diagnosis of Infection with Diarrheagenic *Escherichia coli* and *Shigella* spp. Journal of Clinical Microbiology, v. 42, n. 12, p. 5849–5853, 2004.

GRAPE, M.; SUNDSTRÖM, L.; KRONVALL, G. Sulphonamide resistance gene sul3 found in Escherichia coli isolates from human sources. Journal of Antimicrobial Chemotherapy, v. 52, n. 6, p. 1022-1024, 2003.

JONES, C. H. et al. Characterization and sequence analysis o extended -spectrum- β-lactamase- enconding genes from *Escherichia coli*, *Klebsiella pneumonia* and *Proteus mirabilis* isolates collected during tigecycline phase 3 clinical trials. Antimicrobial Agents and Chemotherapy, v. 1, n. 1, p. 465-475, 2009.

KHAN, S. A.; SUNG, K.; NAWAZ, M. S. Detection of aacA-aphD, qacEδ1, marA, floR, and tetA genes from multidrug-resistant bacteria: comparative analysis of real-time multiplex pcr assays using evagreen® and sybr® green i dyes. Molecular And Cellular Probes, v. 25, n. 2-3, p. 78-86, 2011.

NG, L. K. et al. Multiplex PCR for the detection of tetracycline resistant genes. Molecular and Cellular Probes, v. 15, p. 209-215, 2001.

ODEH, R. et al. Broad resistance due to plasmid-mediated AmpC β-lactamases in clinical isolates of *Escherichia coli*. Clinical Infectious Diseases, v. 35, n. 2, p. 140-145, 2002.
